# Adiposity and risk of ischaemic and haemorrhagic stroke in 0·5 million Chinese men and women: a prospective cohort study

**DOI:** 10.1016/S2214-109X(18)30216-X

**Published:** 2018-05-14

**Authors:** Zhengming Chen, Andri Iona, Sarah Parish, Yiping Chen, Yu Guo, Fiona Bragg, Ling Yang, Zheng Bian, Michael V Holmes, Sarah Lewington, Ben Lacey, Ruqin Gao, Fang Liu, Zengzhi Zhang, Junshi Chen, Robin G Walters, Rory Collins, Robert Clarke, Richard Peto, Liming Li, China Kadoorie Biobank collaborative group, Junshi Chen, Zhengming Chen, Robert Clarke, Rory Collins, Yu Guo, Liming Li, Jun Lv, Richard Peto, Robin Walters, Daniel Avery, Derrick Bennett, Ruth Boxall, Fiona Bragg, Yumei Chang, Yiping Chen, Zhengming Chen, Robert Clarke, Huaidong Du, Simon Gilbert, Alex Hacker, Michael Holmes, Andri Iona, Christiana Kartsonaki, Rene Kerosi, Om Kurmi, Sarah Lewington, Garry Lancaster, Kuang Lin, John McDonnell, Iona Millwood, Qunhua Nie, Jayakrishnan Radhakrishnan, Paul Ryder, Sam Sansome, Dan Schmidt, Paul Sherliker, Rajani Sohoni, Becky Stevens, Iain Turnbull, Robin Walters, Jenny Wang, Lin Wang, Neil Wright, Ling Yang, Xiaoming Yang, Zheng Bian, Ge Chen, Yu Guo, Xiao Han, Can Hou, Jun Lv, Pei Pei, Shuzhen Qu, Yunlong Tan, Canqing Yu, Zengchang Pang, Ruqin Gao, Shaojie Wang, Yongmei Liu, Ranran Du, Yajing Zang, Liang Cheng, Xiaocao Tian, Hua Zhang, Silu Lv, Junzheng Wang, Wei Hou, Jiyuan Yin, Ge Jiang, Xue Zhou, Liqiu Yang, Hui He, Bo Yu, Yanjie Li, Huaiyi Mu, Qinai Xu, Meiling Dou, Jiaojiao Ren, Shanqing Wang, Ximin Hu, Hongmei Wang, Jinyan Chen, Yan Fu, Zhenwang Fu, Xiaohuan Wang, Min Weng, Xiangyang Zheng, Yilei Li, Huimei Li, Yanjun Wang, Ming Wu, Jinyi Zhou, Ran Tao, Jie Yang, Chuanming Ni, Jun Zhang, Yihe Hu, Yan Lu, Liangcai Ma, Aiyu Tang, Shuo Zhang, Jianrong Jin, Jingchao Liu, Zhenzhu Tang, Naying Chen, Ying Huang, Mingqiang Li, Jinhuai Meng, Rong Pan, Qilian Jiang, Weiyuan Zhang, Yun Liu, Liuping Wei, Liyuan Zhou, Ningyu Chen, Hairong Guan, Xianping Wu, Ningmei Zhang, Xiaofang Chen, Xuefeng Tang, Guojin Luo, Jianguo Li, Xiaofang Chen, Xunfu Zhong, Jiaqiu Liu, Qiang Sun, Pengfei Ge, Xiaolan Ren, Caixia Dong, Hui Zhang, Enke Mao, Xiaoping Wang, Tao Wang, Xi Zhang, Ding Zhang, Gang Zhou, Shixian Feng, Liang Chang, Lei Fan, Yulian Gao, Tianyou He, Huarong Sun, Pan He, Chen Hu, Qiannan Lv, Xukui Zhang, Min Yu, Ruying Hu, Hao Wang, Yijian Qian, Chunmei Wang, Kaixue Xie, Lingli Chen, Yidan Zhang, Dongxia Pan, Yuelong Huang, Biyun Chen, Li Yin, Donghui Jin, Huilin Liu, Zhongxi Fu, Qiaohua Xu, Xin Xu, Hao Zhang, Youping Xiong, Huajun Long, Xianzhi Li, Libo Zhang, Zhe Qiu

**Affiliations:** a Clinical Trial Service Unit and Epidemiological Studies Unit, Nuffield Department of Population Health, University of Oxford, UK; b Medical Research Council Population Health Research Unit, Nuffield Department of Population Health, University of Oxford, UK; c Chinese Academy of Medical Sciences, Beijing, China; d Qingdao Centre for Disease Control and Prevention, Qingdao, China; e Suzhou Centre for Disease Control and Prevention, Suzhou, China; f Qingdao Shinan District Centre for Disease Control and Prevention, Shinan District, Qingdao, China; g National Center for Food Safety Risk Assessment, Beijing, China; h Department of Epidemiology and Biostatistics, School of Public Health, Peking University Health Science Center, Beijing, China

## Abstract

**Background:**

China has high stroke rates despite the population being relatively lean. Uncertainty persists about the relevance of adiposity to risk of stroke types. We aimed to assess the associations of adiposity with incidence of stroke types and effect mediation by blood pressure in Chinese men and women.

**Methods:**

The China Kadoorie Biobank enrolled 512 891 adults aged 30–79 years from ten areas (five urban and five rural) during 2004–08. During a median 9 years (IQR 8–10) of follow-up, 32 448 strokes (about 90% confirmed by neuroimaging) were recorded among 489 301 participants without previous cardiovascular disease. Cox regression analysis was used to produce adjusted hazard ratios (HRs) for ischaemic stroke (n=25 210) and intracerebral haemorrhage (n=5380) associated with adiposity.

**Findings:**

Mean baseline body-mass index (BMI) was 23·6 kg/m^2^ (SD 3·2), and 331 723 (67·8%) participants had a BMI of less than 25 kg/m^2^. Throughout the range examined (mean 17·1 kg/m^2^ [SD 0·9] to 31·7 kg/m^2^ [2·0]), each 5 kg/m^2^ higher BMI was associated with 8·3 mm Hg (SE 0·04) higher systolic blood pressure. BMI was positively associated with ischaemic stroke, with an HR of 1·30 (95% CI 1·28–1·33 per 5 kg/m^2^ higher BMI), which was generally consistent with that predicted by equivalent differences in systolic blood pressure (1·25 [1·24–1·26]). The HR for intracerebral haemorrhage (1·11 [1·07–1·16] per 5 kg/m^2^ higher BMI) was less extreme, and much weaker than that predicted by the corresponding difference in systolic blood pressure (1·48 [1·46–1·50]). Other adiposity measures showed similar associations with stroke types. After adjustment for usual systolic blood pressure, the positive associations with ischaemic stroke were attenuated (1·05 [1·03–1·07] per 5 kg/m^2^ higher BMI); for intracerebral haemorrhage, they were reversed (0·73 [0·70–0·77]). High adiposity (BMI >23 kg/m^2^) accounted for 14·7% of total stroke (16·5% of ischaemic stroke and 6·7% of intracerebral haemorrhage).

**Interpretation:**

In Chinese adults, adiposity was strongly positively associated with ischaemic stroke, chiefly through its effect on blood pressure. For intracerebral haemorrhage, leanness, either per se or through some other factor (or factors), might increase risk, offsetting the protective effects of lower blood pressure.

**Funding:**

UK Wellcome Trust, UK Medical Research Council, British Heart Foundation, Cancer Research UK, Kadoorie Charitable Foundation, Chinese Ministry of Science and Technology, Chinese National Natural Science Foundation.

## Introduction

Stroke is a leading cause of premature death and disability worldwide, with more than 80% of the global burden of stroke now occurring in low-income and middle-income countries.^1^ Stroke affects about 2 million people each year in China, and about 20% of cases are due to intracerebral haemorrhage.^2^ The prevalence of overweight and obesity is increasing globally, including in China,^3^, ^4^ and high levels of adiposity are associated with raised blood pressure, raised blood glucose, and dyslipidaemia, each of which predisposes to stroke and ischaemic heart disease.^5^, ^6^, ^7^, ^8^, ^9^ About a fifth of strokes in China has been estimated to be attributable to high adiposity (defined as body-mass index [BMI] >23 kg/m^2^), with no separate estimates available for pathological types of stroke.^10^ Such estimates were based mainly on risk estimates derived from studies done in high-income countries, where, by comparison with China, the mean BMI is higher, the rates of stroke, especially intracerebral haemorrhage, are lower, and individuals identified with hypertension are generally well managed. In China, although several prospective studies have previously assessed the associations between adiposity and stroke mortality, most have predated widespread use of brain imaging for reliable diagnosis of stroke types,^5^, ^8^ which might have important differences in their associations with adiposity.^5^, ^11^

Prospective studies^5^, ^6^, ^8^, ^11^, ^12^, ^13^, ^14^, ^15^, ^16^ have consistently reported positive associations between ischaemic and total stroke and BMI. However, the strength of the associations differs substantially between different populations and, within specific populations, between different studies.^17^ For intracerebral haemorrhage, the previous findings have been conflicting, with some reporting linear positive associations,^8^, ^11^, ^13^, ^18^, ^19^, ^20^ and others showing J-shaped or U-shaped,^5^, ^8^, ^21^ null,^22^, ^23^ or linear inverse associations.^17^ Few studies have assessed the relevance of central (eg, waist circumference) or other (eg, body fat percentage) measures of adiposity with stroke types,^6^, ^24^, ^25^ or the extent to which such effects are mediated by blood pressure, diabetes, or blood lipids.^9^, ^16^ Using data from the large nationwide China Kadoorie Biobank (CKB) prospective study, we assess the associations between general and central adiposity and incidence of stroke types and effect mediation by blood pressure in Chinese men and women.

**Research in context**

**Evidence before this study**

Embase and MEDLINE databases were searched for articles published in English before March 1, 2017, using a combination of terms: “stroke (ischaemic stroke [IS] OR haemorrhagic stroke OR intracerebral haemorrhage [ICH] OR stroke types) AND adiposity (body mass index OR BMI OR waist circumference OR waist hip ratio OR adiposity OR obesity) AND cohort (cohort study OR prospective study OR longitudinal study)”. The reference lists of relevant articles were reviewed. About 20 prospective cohort studies examining the association between adiposity and risk of stroke types were identified, including five in China, along with a few meta-analyses of cohort studies. With a few exceptions, most of the studies only included body-mass index (BMI) and focused on mortality rather than incidence of stroke, with little information on diagnosis of stroke types. Moreover, none examined the quantitative association between BMI and risk of stroke types in the context of the associations between BMI and blood pressure, and between blood pressure and stroke types. In general, these studies have shown positive associations between ischaemic stroke and total stroke and BMI, but the strength of the associations differed substantially between different populations and, within specific populations, between different studies. For intracerebral haemorrhage, the findings have been conflicting, due partly to the relatively small number of events recorded and lack of neuroimaging for diagnosis of stroke types in many studies.

**Added value of this study**

This study represents the world's largest investigation to date in a single study of the association between adiposity and risk of incident stroke types. In this relative lean (mean BMI 23·6 kg/m^2^ [SD 3·2]) and apparently healthy Chinese population, there was a strong positive and log-linear association between general (eg, BMI and body fat percentage) and central (eg, waist circumference and waist-to-hip ratio) adiposity and the incidence of ischaemic stroke, with the risk estimates consistent with those predicted by equivalent differences in systolic blood pressure associated with adiposity. By contrast, the risk estimates for intracerebral haemorrhage were less extreme than for ischaemic stroke, and much weaker than predicted by the corresponding difference in systolic blood pressure, especially at a BMI of less than 25 kg/m^2^, which covers two-thirds of study population. The risk estimates were higher in men than in women, due mainly to stronger associations in men of systolic blood pressure with stroke. After adjustment for usual systolic blood pressure, the positive association of baseline BMI (and other adiposity measures) with ischaemic stroke largely disappeared, whereas for intracerebral haemorrhage it was completely reversed. BMI at the age of 25 years was weakly positively associated with ischaemic stroke and intracerebral haemorrhage, and the association was largely unaffected by adjustment for systolic blood pressure.

**Implications of all the available evidence**

Globally, the mean levels of adiposity have been increasing steadily in recent decades. In this Chinese population, high adiposity (BMI >23 kg/m^2^) accounted for 15% of total stroke (17% ischaemic stroke and 7% intracerebral haemorrhage). The positive association between adiposity and ischaemic stroke is chiefly mediated through its effect on blood pressure. However, there was no association between adiposity and intracerebral haemorrhage across a broad normal range (ie, BMI <25 kg/m^2^), suggesting that leanness, either per se or through some other factor (or factors), might increase risk, thereby offsetting the protective effects of lower blood pressure. Future Mendelian randomisation studies are needed to establish the causal relevance of adiposity for intracerebral haemorrhage. Since ischaemic stroke constitutes the majority of total stroke cases in China and elsewhere, the findings on intracerebral haemorrhage should not diminish the fundamental importance of high adiposity as a major modifiable determinant of overall stroke. Given the substantial risks associated with high blood pressure, the present study further highlights the importance of controlling blood pressure (and other intermediate risk factors) for prevention of both ischaemic and haemorrhagic stroke, irrespective of levels of adiposity.

## Methods

### Study population

Details of the design, methods, and participants in the CKB have been described previously.^26^, ^27^, ^28^ Briefly, the baseline survey took place between June 25, 2004, and July 15, 2008, involving ten (five urban and five rural) areas across China, chosen from China's nationally representative Disease Surveillance Points to maximise geographical and social diversity. About 1·8 million registered residents aged 35–74 years in the 100–150 administrative units (rural villages or urban residential committees) within each of the ten study areas were identified through residential records and invited to participate in the study. 512 891 people, including 12 668 just outside this age range (extending the baseline age range to 30–79 years), were enrolled in the study. Prior ethics approval was obtained from the relevant international, national, and local ethics committees. All participants provided written informed consent.

### Data collection

Information was collected on sociodemographic factors, lifestyle (eg, smoking, alcohol consumption, diet, and physical activity), and medical history by trained health workers using interviewer-administered laptop-based questionnaires. Physical measurements (eg, blood pressure, lung function, and anthropometric measures) were recorded using standard methods. A non-fasting venous blood sample was collected for storage and on-site random plasma glucose testing using the SureStep Plus system (LifeScan; Milpitas, CA, USA). Two periodic resurveys were done from May 26, to Oct 10, 2008, and from Aug 4, 2013, to Sept 18, 2014, on approximately 5% of randomly selected participants, collecting similar information as at baseline to enable assessment of within-person variation and temporal trends in exposures.

Anthropometric measurements were recorded once, and usually to the nearest 0·1 cm or 0·1 kg.^27^, ^28^ Standing and sitting heights were measured using a stadiometer, and weight was measured by a body composition analyser (TANITA-TBF-300 GS; Tanita Corporation, Tokyo, Japan), with subtraction of weight of clothing by 0·5–1·5 kg depending on the season. Body fat percentage was the proportion of total weight due to fat weight estimated by the body composition analyser. Waist circumference was measured, using a soft non-stretchable tape, midway between the lowest rib and the iliac crest. Hip circumference was measured at the maximum circumference around the buttocks (usually over underclothing, but subtracting 1 cm if over a skirt, or 2·5 cm if over trousers). BMI was calculated as measured weight in kilograms divided by the square of the standing height in metres (kg/m^2^), BMI at age 25 years used self-reported weight at age 25 years and height measured at baseline, and waist-to-hip ratio was the ratio of waist circumference to hip circumference.

Blood pressure was measured twice using the UA-779 digital sphygmomanometer (A&D Instruments; Abingdon, UK) after participants had remained at rest in the seated position for at least 5 min. If the difference between the two measurements was more than 10 mm Hg for systolic blood pressure (in about 5% of participants), a third measurement was made and the mean of the last two measurements was used.

### Follow-up for morbidity and mortality

The vital status of participants was obtained periodically from local death registries,^29^ and checked annually against residential records and health insurance data and by active confirmation through local residential administrators. Causes of death were sought chiefly from official death certificates. Additional information on hospitalised events was collected through electronic linkage, via unique personal identification numbers, to disease registries (for stroke, ischaemic heart disease, cancer, and diabetes) and to the national health insurance claim system, which has almost universal coverage and included disease descriptions, International Classification of Diseases 10th revision (ICD-10) codes, and procedure codes.^27^ All reported fatal or non-fatal stroke (and other major diseases) events from different sources were reviewed and integrated centrally by trained clinical staff, who were masked to baseline information.

The main stroke types examined were ischaemic stroke (ICD-10 code I63), intracerebral haemorrhage (I61), subarachnoid haemorrhage (I60), and total stroke (I60, I61, I63, and I64). All analyses were restricted to first events of any stroke type occurring between the ages of 35 years and 79 years, with censoring when participants had a stroke or reached 80 years of age (or loss to follow-up).

### Statistical analyses

Individuals with previous history of stroke or transient ischaemic attack (n=8884) or ischaemic heart disease (n=15 472) at baseline, and those with missing, implausible, or extreme values of anthropometric measurements (n=493) were excluded, leaving 489 301 for our report. Individuals with hypertension or diabetes were retained (and not adjusted for in standard analyses), as these could be mechanisms whereby adiposity predisposes to stroke.

The prevalence or mean values of baseline characteristics were calculated across seven BMI categories (cut points <18·0, <20·5, <23·0, <25·0, <27·5, and <30·0 kg/m^2^), standardised by 5-year age groups, sex, and study area. The cut points were chosen to cover different thresholds for defining overweight and obesity in Chinese people and to ensure reasonable participant numbers in each group. Cox proportional hazards models with time since baseline as the timescale were used to estimate hazard ratios (HRs) for incident stroke by adiposity and systolic blood pressure, stratified by 5-year age-at-risk, sex, and area (ten groups), and adjusted for education (five groups), smoking (four groups), alcohol use (five groups), physical activity (metabolic equivalent of task [MET] hours per day), and self-reported health status (four groups). Floating variance estimates (reflecting independent variability within each group, including the reference group) were used to estimate group-specific 95% CIs for each log HR to enable comparisons between any two categories (rather than just pairwise comparisons with the reference category).^30^

To correct for regression dilution bias,^31^ two serial measurements recorded in a random subset of approximately 20 000 participants at resurveys were used to calculate regression dilution ratios (ie, the ratio of the uncorrected to the corrected regression coefficient). Log HR estimates associated with baseline measures were divided by the regression dilution ratio (0·61 for systolic blood pressure and 0·93 for BMI) to obtain HRs for usual levels of exposure measures.

Departure from linearity was assessed using the likelihood ratio test, with inclusion of a second-degree term in the model.^32^ The proportional hazards assumption was tested by examining the HRs for the first 4 years and for subsequent years of follow-up (and showed no strong evidence of departure). HRs were also compared across strata of other baseline factors (eg, age, sex, and area), and χ^2^ values for trend and heterogeneity were calculated. Additional sensitivity analyses, presented mainly in the appendix, were done after excluding individuals with poor self-rated health status, any self-reported previous disease at baseline, ever smokers, or stroke events occurring during the first 3-years of follow-up. All analyses were done with SAS version 9.3.

### Role of the funding source

The funders of the study had no role in study design, data collection, data analysis, data interpretation, or writing of the report. ZC, AI, and LL had full access to all the data in the study and had final responsibility for the decision to submit for publication.

## Results

Among the 489 301 participants included, the mean age was 51·1 years (SD 10·4), and the mean BMI was 23·6 kg/m^2^ (3·2), with 331 723 (67·8%) having a BMI of less than 25 kg/m^2^ and 18 862 (3·9%) having a BMI of 30 kg/m^2^ or more (ie, obese). Overall mean BMI was slightly higher in women than in men (23·7 kg/m^2^ [3·4] *vs* 23·4 kg/m^2^ [3·2]; p<0·0001), and higher in urban than in rural areas (24·2 kg/m^2^ [3·4] *vs* 23·1 kg/m^2^ [3·3]; p<0·0001). BMI was weakly positively associated with education (p=0·0008) and alcohol intake (p=0·0009), especially at a BMI of less than 25 kg/m^2^, and inversely associated with smoking in both men and women. Individuals with a BMI of less than 18 kg/m^2^ or more than 25 kg/m^2^ tended to have lower levels of physical activity and poor self-rated health status than did those with a normal BMI (table 1).

**Table 1:** Baseline characteristics of participants by baseline body-mass index among people without previous stroke or ischaemic heart disease

		**Categories of baseline body-mass index (kg/m^2^)**							**All**
		<18·0	18·0 to <20·5	20·5 to <23·0	23·0 to <25·0	25·0 to <27·5	27·5 to <30·0	≥30·0	
Number of participants		13 527	72 411	135 616	110 169	95 573	43 143	18 862	489 301
Body-mass index		17·1 (0·9)	19·5 (0·8)	21·8 (0·7)	23·9 (0·6)	26·1 (0·7)	28·5 (0·8)	31·7 (2·0)	23·6 (3·2)
Age and socioeconomic factors									
	Age (years)	55·4 (15·1)	51·2 (11.9)	50·4 (10·6)	50·7 (10·1)	51·0 (10·1)	51·1 (10·5)	51·0 (12·2)	51·1 (10·4)
	Male	37·8%	45·3%	42·1%	40·2	40·0%	37·5%	29·1%	40·9%
	Female	62·2%	54·7%	57·9%	59·8%	60·0%	62·5%	70·9%	59·1%
	Urban residents	31·7%	32·0%	38·4%	45·5%	50·5%	53·9%	57·2%	43·2%
	Rural residents	68·3%	68·0%	61·6%	54·5%	49·5%	46·1%	42·8%	56·8%
	≥6 years of education	48·1%	48·8%	49·4%	50·2%	49·9%	48·9%	47·6%	49·2%
Lifestyle factors									
	Male ever-regular smoker	79·7%	79·7%	75·9%	72·5%	70·9%	70·5%	70·7%	74·4%
	Female ever-regular smoker	5·4%	3·7%	3·1%	2·9%	2·7%	2·8%	3·2%	3·1%
	Male ever-regular alcohol drinker	31·8%	36·1%	37·8%	37·6%	37·0%	37·7%	36·2%	37·2%
	Female ever-regular alcohol drinker	2·4%	2·3%	2·5%	2·5%	2·6%	2·6%	2·2%	2·5%
	Physical activity (MET h/day)	20·7 (17·1)	22·1 (13·0)	22·1 (11·9)	21·6 (11·8)	21·0 (12·2)	20·4 (13·0)	19·5 (15·1)	21·5 (11·8)
Medical history and health status									
	Diabetes	3·6%	3·2%	4·2%	5·5%	6·8%	8·1%	10·4%	5·4%
	Hypertension*	2·8%	4·3%	6·7%	10·1%	14·0%	18·1%	23·9%	9·9%
	Self-rated poor health	19·1%	11·5%	9·0%	8·2%	8·4%	9·4%	11·6%	9·4%
Anthropometry and blood pressure									
	Weight (kg)	43·2 (5·3)	49·1 (4·4)	54·9 (4·3)	60·4 (4·4)	65·9 (5·0)	72·1 (5·9)	80·3 (9·0)	59·1 (9·1)
	Standing height (cm)	158·6 (8·4)	158·6 (6·3)	158·6 (5·6)	158·7 (5·4)	158·9 (5·6)	158·9 (6·1)	159·0 (7·2)	158·7 (5·5)
	Sitting height (cm)	84·4 (4·8)	84·7 (3·7)	85·0 (3·3)	85·4 (3·1)	85·7 (3·2)	86·0 (3·5)	86·3 (4·0)	85·3 (3·2)
	Waist circumference (cm)	64·8 (6·4)	69·9 (5·5)	75·6 (5·1)	81·1 (5·0)	86·3 (5·4)	91·8 (6·2)	98·8 (8·5)	80·0 (9·0)
	Waist-to-hip ratio	0·80 (0·07)	0·83 (0·06)	0·86 (0·05)	0·89 (0·05)	0·91 (0·06)	0·94 (0·06)	0·96 (0·07)	0·88 (0·06)
	Body fat percentage	15·8 (4·0)	20·3 (3·2)	24·8 (3·3)	28·8 (3·5)	32·4 (4·1)	36·0 (4·9)	40·0 (6·8)	27·9 (6·4)
	Systolic blood pressure (mm Hg)	119·8 (24·3)	123·8 (21·3)	127·7 (19·1)	131·2 (18·9)	134·8 (19·8)	138·4 (22·0)	142·7 (26·4)	130·6 (19·3)
	Diastolic blood pressure (mm Hg)	72·7 (14·4)	74·0 (11·7)	75·8 (10·6)	78·0 (10·5)	80·1 (11·1)	82·0 (12·3)	83·9 (14·8)	77·7 (10·7)
Random plasma glucose concentration (mmol/L)		5·90 (3·88)	5·81 (2·56)	5·89 (2·29)	6·02 (2·27)	6·18 (2·46)	6·33 (2·71)	6·61 (3·79)	6·03 (2·23)
Lipid concentration									
	Data available	653	2929	4850	3731	3491	1712	773	18 139
	LDL cholesterol (mmol/L)	2·05 (0·82)	2·20 (0·76)	2·30 (0·68)	2·44 (0·68)	2·49 (0·69)	2·54 (0·78)	2·59 (1·08)	2·37 (0·67)
	HDL cholesterol (mmol/L)	1·42 (0·43)	1·35 (0·37)	1·28 (0·31)	1·21 (0·27)	1·15 (0·27)	1·12 (0·27)	1·11 (0·30)	1·24 (0·29)
	Total cholesterol (mmol/L)	4·32 (1·15)	4·46 (1·01)	4·57 (0·95)	4·74 (0·95)	4·81 (0·97)	4·88 (1·08)	4·95 (1·53)	4·66 (0·94)
	Triglycerides (mmol/L)	1·19 (0·77)	1·44 (1·40)	1·72 (1·28)	2·09 (1·73)	2·45 (1·93)	2·71 (2·18)	2·84 (2·32)	2·00 (1·57)

Baseline characteristics adjusted for age, sex, and area as appropriate. Data are mean (SD) and weighted percentages. MET h/day=metabolic equivalent of task hours per day.

* When combined self-reported hypertension with measured high blood pressure (ie, ≥140/90 mm Hg), 159 377 (32·6%) participants had hypertension at baseline.

BMI was strongly positively associated with weight (p<0·0001), and weakly positively associated with sitting and standing height (p=0·0006; table 1; appendix p 3). BMI, waist circumference, waist-to-hip ratio, and body fat percentage were highly intercorrelated with each other, and BMI at the age of 25 years was modestly correlated with adulthood BMI and less so with other adiposity measures (appendix p 3). BMI, and other measures of adiposity,^28^ were strongly positively associated with blood pressure and prevalence of physician-diagnosed hypertension and diabetes (table 1). BMI was also positively correlated with plasma glucose concentration. In a subset of 18 139 participants not receiving lipid-lowering treatment, BMI was positively correlated with concentrations of LDL cholesterol, total cholesterol, and triglycerides, and inversely correlated with HDL cholesterol (table 1).

Incident strokes from enrolment until Jan 1, 2016 (a median of 9 years [IQR 8–10]), are included in these analyses, by which time 37 289 (7·3%) participants had died and 4875 (1·0%) were lost to follow-up. During 3·85 million person-years of follow-up, there were 32 448 incident cases of stroke (about 90% confirmed by neuroimaging; 3493 [10·8%] fatal) at ages 35–79 years, including 25 210 (77·7%) due to ischaemic stroke (mean age at event 65·1 years [SD 9·6]; 648 [2·6%] fatal), 5380 (16·6%) due to intracerebral haemorrhage (mean age at event 64·6 years [10·4]; 2495 [46·4%] fatal), 434 (1·3%) due to subarachnoid haemorrhage (mean age at event 61·4 years [10·7]; 43 [9·9%] fatal), and 1424 (4·4%) unspecified strokes (mean age at event 65·0 years [10·1]; 307 [21·6%] fatal). The standardised total stroke incidence per 10 000 person-years was 84·3, and was higher in urban than rural areas (94·6 *vs* 76·8). The incidence of ischaemic stroke per 10 000 person-years was higher in urban than in rural areas (82·2 *vs* 53·3), but the converse was true for intracerebral haemorrhage (7·5 *vs* 18·7).

**Figure 1:**
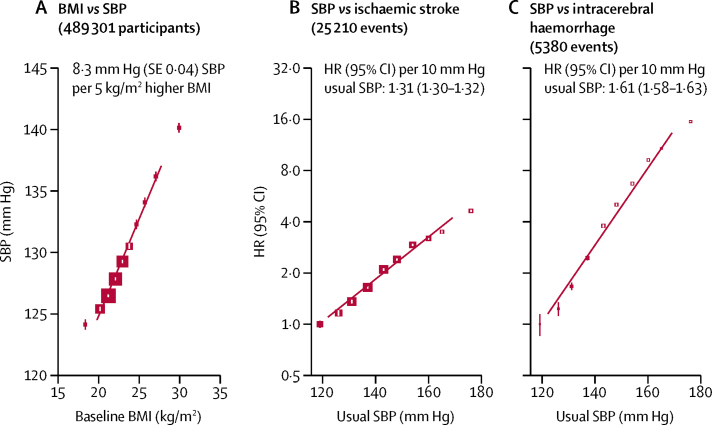
Associations between baseline BMI and SBP and between usual SBP and incidence of stroke types (A) BMI versus SBP. Means are adjusted for age, sex, area, education, smoking, alcohol consumption, physical activity, and self-rated health status. The area of the squares is inversely proportional to the variance of the mean SBP. The vertical lines indicate 95% CIs. (B) SBP versus ischaemic stroke. (C) SBP versus intracerebral haemorrhage. HRs are stratified by age, sex, and area, and adjusted simultaneously for education, smoking, alcohol consumption, physical activity, and self-rated health status. Each closed square represents HR with the area inversely proportional to the variance of the log HR. The vertical lines indicate 95% CIs. BMI=body-mass index. SBP=systolic blood pressure. HR=hazard ratio.

Throughout the distribution, each 5 kg/m^2^ higher BMI was associated with 8·3 mm Hg (SE 0·04) higher systolic blood pressure (and 4·3 mm Hg [0·02] higher diastolic blood pressure; figure 1). Blood pressure was strongly positively associated with risk of stroke, especially intracerebral haemorrhage, with no evidence of any threshold throughout the range studied (figure 1). After adjusting for regression dilution bias, each 10 mm Hg higher usual systolic blood pressure was associated with an adjusted HR of 1·31 (95% CI 1·30–1·32) for ischaemic stroke and 1·61 (1·58–1·63) for intracerebral haemorrhage, with greater HRs at younger than older ages and in men than women (appendix p 4). Corresponding adjusted HRs per 10 mm Hg higher systolic blood pressure were 1·21 (1·13–1·30) for subarachnoid haemorrhage, 1·38 (1·33–1·43) for unspecified stroke, and 1·36 (1·36–1·37) for total stroke. Given the linear associations in figure 1, it can be expected that a 5 kg/m^2^ higher BMI, corresponding to 8·3 mm Hg higher usual systolic blood pressure, would be associated with HRs of 1·25 (1·24–1·26) for ischaemic stroke, 1·48 (1·46–1·50) for intracerebral haemorrhage, and 1·29 (1·29–1·30) for total stroke.

**Figure 2:**
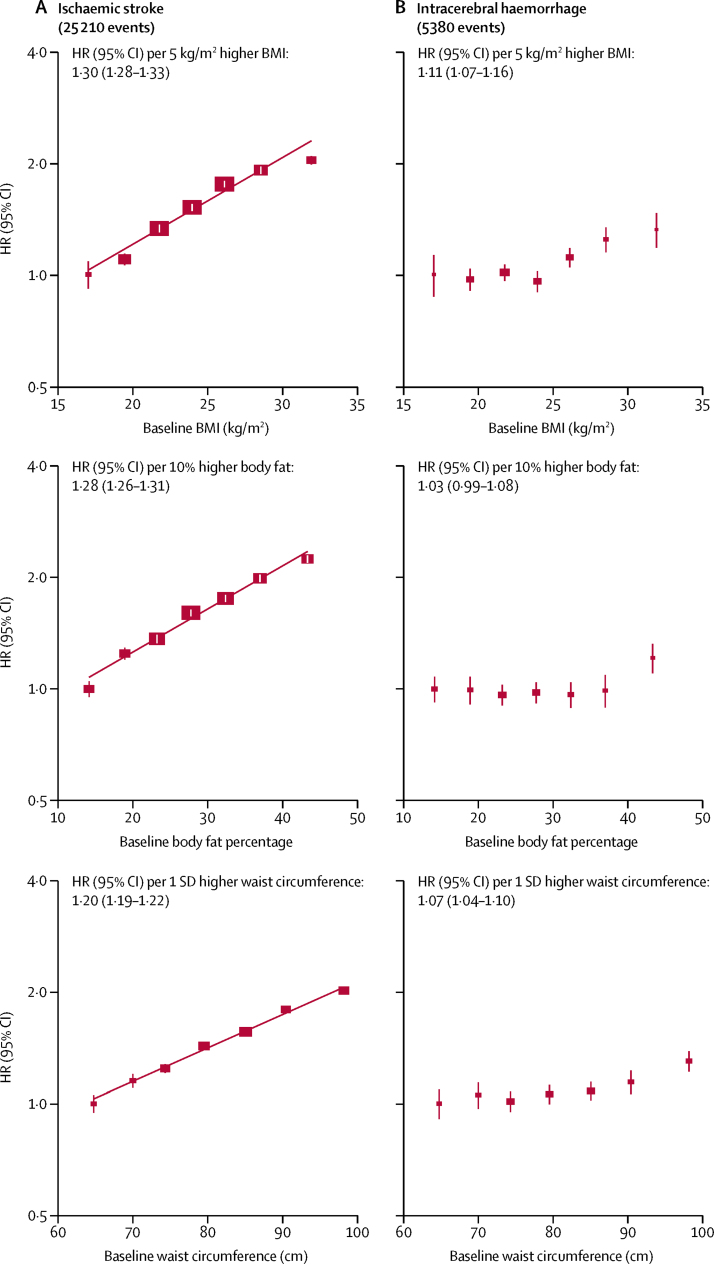
Adjusted HRs for ischaemic stroke (A) and intracerebral haemorrhage (B) by baseline BMI, body fat percentage, and waist circumference HRs are stratified by age, sex, and area, and adjusted simultaneously for education, smoking, alcohol consumption, physical activity, and self-rated health status. Each closed square represents HR with the area inversely proportional to the variance of the log HR. The vertical lines indicate 95% CIs. HR=hazard ratio. BMI=body-mass index.

There was a log-linear positive association between BMI and incidence of ischaemic stroke throughout the BMI range examined (mean 17·1 kg/m^2^ [SD 0·9] to 31·7 kg/m^2^ [2·0]), with each 5 kg/m^2^ higher baseline BMI associated with an adjusted HR of 1·30 (95% CI 1·28–1·33; table 2; figure 2), consistent with the expected associations with systolic blood pressure in figure 1. By contrast, each 5 kg/m^2^ higher BMI was only associated with an adjusted HR of 1·11 (1·07–1·16) for intracerebral haemorrhage, an effect size that was weaker than that for ischaemic stroke, and substantially weaker than predicted from the corresponding difference in systolic blood pressure (ie, 1·48 [1·46–1·50] per 8·3 mm Hg systolic blood pressure). Moreover, the shape of the association with intracerebral haemorrhage appeared non-linear (p<0·0001 for test of non-linearity). Among the 68% of participants with a BMI of less than 25 kg/m^2^, there were no associations between BMI and risk of intracerebral haemorrhage, even though systolic/diastolic blood pressure differed by 11·5/5·3 mm Hg across the four BMI groups studied. Above a BMI of 25 kg/m^2^, the risk of intracerebral haemorrhage increased with increasing levels of BMI, but the association was still weaker than that predicted by the difference in systolic blood pressure. BMI was unrelated to subarachnoid haemorrhage, but the number of events (n=434) was small (table 2). For unspecified stroke, the association resembled that for ischaemic stroke (HR 1·28 [1·18–1·38] per 5 kg/m^2^), as was the case for total stroke (1·27 [1·25–1·29] per 5 kg/m^2^; table 2). After adjusting for regression dilution bias, the HRs became slightly stronger (1·33 [1·30–1·36] for ischaemic stroke, 1·12 [1·07–1·17] for intracerebral haemorrhage, and 1·29 [1·27–1·31] for total stroke, per 5 kg/m^2^ higher usual BMI). In this population, high adiposity (defined as BMI >23 kg/m^2^) accounted for 14·7% of total stroke, including 16·5% of ischaemic stroke, and 6·7% of intracerebral haemorrhage, and overweight (ie, BMI ≥25 kg/m^2^) accounted for 9·2% of total stroke, including 9·9% of ischaemic stroke and 6·7% of intracerebral haemorrhage (appendix p 5).

**Table 2:** Standardised stroke incidence rates per 10 000 person-years and adjusted HRs by baseline BMI among people without previous stroke or ischaemic heart disease

		**Ischaemic stroke**			**Intracerebral haemorrhage**			**Subarachnoid haemorrhage**			**Unspecified stroke**			**All stroke**		
		Events	Rate*	HR (95% CI)†	Events	Rate*	HR (95% CI)†	Events	Rate*	HR (95% CI)†	Events	Rate*	HR (95% CI)†	Events	Rate*	HR (95% CI)†
BMI<18·0		590	49·3	1·00 (0·92–1·09)	253	15·8	1·00 (0·88–1·13)	10	1·2	1·00 (0·53–1·87)	37	2·3	1·00 (0·72–1·39)	890	68·6	1·00 (0·94–1·07)
BMI 18·0–20·4		2740	50·4	1·10 (1.05–1·14)	980	13·5	0·97 (0·91–1·03)	71	1·1	1·53 (1·20–1·94)	207	3·5	1·41 (1·23–1·63)	3998	68·5	1·08 (1·04–1·11)
BMI 20·5–22·9		5790	59·6	1·33 (1·29–1·36)	1494	13·7	1·01 (0·96–1·06)	127	1·3	1·57 (1·32–1·87)	349	3·4	1·47 (1·32–1·63)	7760	78·0	1·24 (1·22–1·27)
BMI 23·0–24·9		5600	67·5	1·51 (1·47–1·55)	1020	12·9	0·95 (0·90–1·02)	81	1·0	1·25 (1·01–1·56)	306	3·8	1·61 (1·44–1·80)	7007	85·2	1·37 (1·33–1·40)
BMI 25·0–27·4		5946	78·6	1·75 (1·70–1·79)	960	14·6	1·11 (1·04–1·18)	89	1·2	1·57 (1·28–1·94)	289	4·0	1·75 (1·56–1·97)	7284	98·4	1·58 (1·54–1·62)
BMI 27·5–29·9		3038	86·6	1·90 (1·84–1·97)	464	17·7	1·24 (1·13–1·36)	43	1·3	1·66 (1·22–2·25)	150	4·5	2·03 (1·73–2·39)	3695	110·1	1·73 (1·68–1·79)
BMI≥30·0		1506	96·7	2·03 (1·92–2·13)	209	19·5	1·32 (1·15–1·51)	13	1·2	1·05 (0·60–1·82)	86	6·9	2·60 (2·09–3·22)	1814	124·3	1·85 (1·76–1·94)
Overall		25 210	65·5	..	5380	14·0	..	434	1·1	..	1424	3·7	..	32 448	84·3	..
p_trend_		..	..	<0·0001	..	..	<0·0001‡	..	..	0·9686	..	..	<0·0001	..	..	<0·0001
HRs per 5 kg/m^2^																
	Observed	..	..	1·30 (1·28–1·33)	..	..	1·11 (1·07–1·16)	..	..	0·96 (0·83–1·11)	..	..	1·28 (1·18–1·38)	..	..	1·27 (1·25–1·29)
	Expected¶	..	..	1·25 (1·24–1·26)	..	..	1·48 (1·46–1·50)	..	..	1·17 (1·11–1·24)	..	..	1·31 (1·27–1·35)	..	..	1·29 (1·29–1·30)

Body-mass index (BMI) unit is kg/m^2^. HR=hazard ratio. ..=not applicable.

* Per 10 000 person-years. Standardised to age, sex, and study area structure of the China Kadoorie Biobank population.

† Stratified by age, sex, and study area and adjusted for education, smoking, alcohol consumption, physical activity, and self-rated health status.

‡ p value <0·0001 both for trend test and for test of non-linearity across seven groups.

¶ Estimated according to the corresponding difference in usual systolic blood pressure (8·3 mm Hg) and its associated stroke risk.

**Figure 3:**
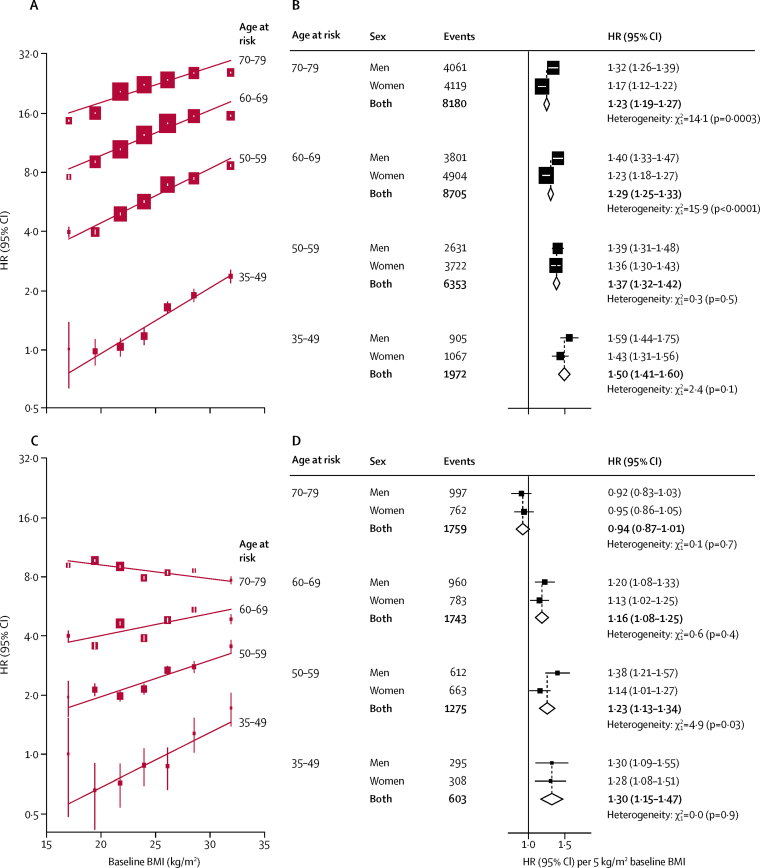
Adjusted HRs for ischaemic stroke (A,B) and intracerebral haemorrhage (C,D) by baseline BMI, stratified by age (A,C) and age and sex (B,D) The left panel shows adjusted HRs for stroke types by baseline BMI, stratified by age-at-risk. HRs are stratified by sex and area, and adjusted simultaneously for education, smoking, alcohol consumption, physical activity, and self-rated health status. HRs are plotted on a floating absolute scale. Each closed square represents HR with the area inversely proportional to the variance of the log HR. The vertical lines indicate 95% CIs. The right panel shows HRs for stroke types per 5 kg/m^2^ higher BMI, stratified by age-at-risk and sex. Each square represents HR with the area inversely proportional to the variance of the log HR. The horizontal lines indicate 95% CIs. The dashed vertical line indicates the overall HR for men and women combined, and open diamonds indicate combined values and their 95% CIs. HR=hazard ratio. BMI=body-mass index.

The HRs for ischaemic stroke per 5 kg/m^2^ baseline BMI were greater at younger than at older ages (figure 3). The HRs were also greater in men than women (1·39 [95% CI 1·35–1·43] *vs* 1·26 [1·23–1·28]; p_heterogeneity_<0·0001), especially at age-at-risk of more than 60 years. For intracerebral haemorrhage, there was a weak inverse association with BMI at ages 70–79 years but, at younger than 70 years, BMI was weakly and positively associated with intracerebral haemorrhage.

The associations between BMI and ischaemic stroke and intracerebral haemorrhage were similar in urban and rural areas (appendix p 6) and across the ten study areas (appendix p 7). The associations were unaltered by smoking status (appendix p 8), and by other factors except education (p_heterogeneity_=0·035 for ischaemic stroke and p_heterogeneity_=0·0038 for intracerebral haemorrhage) and, to a lesser extent, by physical activity (p_trend_<0·0001 for ischaemic stroke and p_trend_=0·088 for intracerebral haemorrhage; appendix p 9). These associations were unaltered by additional adjustment for certain dietary factors (eg, fresh fruit, preserved vegetable, dairy, and meat intake; HR 1·31 [95% CI 1·29–1·33] for ischaemic stroke and 1·12 [1·08–1·17] for intracerebral haemorrhage, per 5 kg/m^2^). The associations were slightly attenuated by additional exclusion of people with any previous diseases, people with self-reported poor health status at baseline, the first 3 years of follow-up, and ever-regular smokers (appendix p 10), but there was still no association with intracerebral haemorrhage at a BMI of less than 25 kg/m^2^.

The associations were, however, substantially attenuated by additional adjustment for blood pressure. For ischaemic stroke, simultaneous adjustment for usual systolic blood pressure (and diabetes status) reduced the HR from 1·30 (95% CI 1·28–1·33) to 1·05 (1·03–1·07; appendix p 11), whereas for intracerebral haemorrhage, the association was reversed (from 1·11 [1·07–1·16] to 0·73 [0·70–0·77]), including among individuals at age 35–49 years (0·66 [0·58–0·75]). There was no longer any sex difference in the strength of the associations between BMI and risk of ischaemic stroke and intracerebral haemorrhage after adjustment for usual systolic blood pressure. Likewise, the results were unaltered in the subset of participants after additional adjustment for LDL cholesterol, HDL cholesterol, and triglycerides (1·03 [0·99–1·08] for ischaemic stroke and 0·83 [0·78–0·87] for intracerebral haemorrhage, per 5 kg/m^2^).

The shape and strength of the associations with other measures of adiposity (eg, body fat percentage and waist circumference) were generally similar to those for BMI (figure 2), as were the attenuating effects of adjustment for mediators (appendix pp 11–12) and augmenting effects of adjustment for regression dilution bias (appendix p 13). BMI at the age of 25 years was weakly positively associated with ischaemic stroke and intracerebral haemorrhage, and the association was largely unaffected by adjustment for systolic blood pressure (appendix p 14). BMI change between the age of 25 years and the baseline survey was also positively associated with ischaemic stroke, but the association disappeared almost completely after adjustment for systolic blood pressure, whereas for intracerebral haemorrhage, there was no association without adjustment and a strongly inverse association after adjustment for systolic blood pressure (appendix p 14).

## Discussion

This large prospective study showed that, among relatively lean but healthy Chinese adults, adiposity was strongly positively associated with systolic blood pressure, and that systolic blood pressure was strongly positively related to stroke incidence, particularly intracerebral haemorrhage, without evidence of any threshold throughout the range studied. However, despite these two continuous and apparently causal relationships, adiposity showed an expected strong positive association only with ischaemic stroke, but not with intracerebral haemorrhage.

Several large prospective studies, and meta-analyses of such studies, have consistently reported positive associations between BMI and risks of ischaemic stroke and total stroke.^5^, ^6^, ^8^, ^11^, ^12^, ^13^, ^15^, ^16^, ^17^ In a meta-analysis of 16 large prospective studies, including eight from east Asia, the pooled relative risk (RR) for ischaemic stroke was greater in east Asian than in high-income populations (1·35 *vs* 1·22, per 5 kg/m^2^ higher BMI),^17^ but varied greatly across different studies both within and between east Asian and high-income populations. Our RR estimate for ischaemic stroke (1·30 per 5 kg/m^2^ BMI) was similar to that observed in previous east Asian studies. Likewise, a meta-analysis of 97 prospective studies, including 33 from Asia, which examined the associations of BMI with total stroke only, reported pooled RR in Asian cohorts that were similar to those reported in the present study (1·29 *vs* 1·27 per 5 kg/m^2^ higher BMI), but stronger than those in high-income populations (1·14).^16^ For total stroke, our study estimated that high BMI (defined as >23 kg/m^2^) accounted for 15% of total stroke, which was less than the 20% previously estimated for China in a recent Global Burden of Disease report^10^ that used the same BMI cut points. Moreover, our study also demonstrated that the RRs for ischaemic stroke were substantially greater at younger than older ages, although the absolute excess risks were greater at older ages. Previous studies showed that, given BMI levels, men tend to have greater insulin resistance, ectopic fat levels in the liver and elsewhere, and higher risks of type 2 diabetes and cardiovascular disease.^9^, ^33^ In our study, the RRs were also higher in men than in women, which was due mainly to the stronger associations in men between systolic blood pressure and stroke. Consistent with previous studies,^6^, ^16^ the observed association of BMI with ischaemic stroke was mediated mainly through blood pressure in this Chinese population.

Most previous studies have focused mainly on haemorrhagic stroke rather than intracerebral haemorrhage specifically, and the results have been inconsistent, partly because of the relatively small number of cases studied and the strong possibility of misclassification of stroke types in studies without widespread use of brain imaging. In the most recent meta-analysis, which included more than 17 000 haemorrhagic stroke cases,^17^ the overall association between BMI and risk of haemorrhagic stroke differed substantially between high-income and east Asian cohorts (RR 0·91 *vs* 1·16, per 5 kg/m^2^ higher BMI). In the high-income cohorts, more than 80% of the haemorrhagic stroke cases were from one UK study, which used self-reported BMI and had a disproportionally large number of subarachnoid haemorrhage cases compared with intracerebral haemorrhage cases (3062 *vs* 2790). Moreover, this UK study reported no increased risk of intracerebral haemorrhage even among those who were obese, nor a positive association with risk of ischaemic stroke at BMI levels of less than 27·5 kg/m^2^, in contrast with the findings of this and many other previous studies.^5^, ^6^, ^11^, ^12^, ^13^, ^16^

Consistent with our study, several large prospective studies in east Asian populations have reported little or no clear positive associations between BMI and risk of haemorrhagic stroke at a BMI of less than 25 kg/m^2^.^5^, ^8^, ^11^, ^12^ However, none of the previous studies included sufficient numbers of well characterised intracerebral haemorrhage cases as in this study, or attempted to quantify the associations in the context of the association between adiposity and systolic blood pressure and between systolic blood pressure and stroke risk. Among the two-thirds of CKB participants with a BMI of less than 25 kg/m^2^, there was no association between adiposity and intracerebral haemorrhage. Given the strong positive associations observed between adiposity and systolic blood pressure and between systolic blood pressure and intracerebral haemorrhage, the lack of any apparent association with intracerebral haemorrhage below BMI 25 kg/m^2^ was unexpected and unexplained. There was a mean difference of at least 11/5 mm Hg in systolic/diastolic blood pressure across the BMI range below 25 kg/m^2^, and this difference should, other things being equal, correspond to an HR of 1·6 for intracerebral haemorrhage, but it was totally flat. The disparities are too extreme to be accounted for by chance, by known confounding factors, or by reverse causality, although one cannot exclude the possibility that some other unmeasured or unknown risk factors associated with low BMI might offset the protective effects of lower blood pressure. Future Mendelian randomisation studies are needed to establish the causal relevance of adiposity for intracerebral haemorrhage.^34^

Few prospective cohort studies have examined the associations between stroke types and measures of adiposity other than BMI, such as waist circumference, waist-to-hip ratio, and body fat percentage.^6^, ^24^, ^25^ Compared with raised BMI, measures of central adiposity, such as waist circumference and waist-to-hip ratio, might be better indicators of accumulation of visceral fat and an adverse metabolic profile.^35^, ^36^, ^37^ However, in the Emerging Risk Factors Collaboration meta-analysis of 21 studies involving about 2400 ischaemic stroke cases,^6^ BMI, waist circumference, and waist-to-hip ratio each showed a similar strength of association with ischaemic stroke risk. This study included a ten times greater number of ischaemic stroke cases than did the latter meta-analysis and confirmed the similar associations between ischaemic stroke and different measures of adiposity. These associations are consistent with the fact that most of the adiposity measures (eg, BMI and waist circumference) were highly correlated with each other. Moreover, this study also provided a new assessment of the relevance of central adiposity and other measures of adiposity (eg, BMI at age 25 years and body fat percentage) for intracerebral haemorrhage.

Our study had several strengths, including a very large study population, availability of different measures of adiposity, completeness of follow-up, and a high proportion of stroke types reliably diagnosed by neuroimaging. Moreover, this study explored the effects of mediators (eg, systolic blood pressure) on the associations of adiposity with stroke types both directly and indirectly. However, the study also had several limitations. First, despite the widespread use of neuroimaging, the study was not able to examine the associations between adiposity and different subtypes of ischaemic stroke (eg, lacunar *vs* non-lacunar ischaemic stroke) or of intracerebral haemorrhage (eg, lobar *vs* non-lobar intracerebral haemorrhage). Second, information collected on diet was limited and did not include salt intake, so residual confounding might still persist, even though adjustment for fresh fruit and meat consumption did not alter the associations. Third, it was not possible to fully explore the mediating effects of lipids and other blood-related factors, although examination of a subset of data indicated that further adjustment for lipids had little additional impact over and above blood pressure.

In summary, our study shows that, among relatively lean but healthy Chinese adults, adiposity was strongly and positively associated with ischaemic stroke, mainly through its effect on blood pressure. However, there was no association between adiposity and intracerebral haemorrhage across the normal range (ie, BMI <25 kg/m^2^), suggesting that leanness, either per se or through some other factor (or factors), might increase risk, thereby offsetting the protective effects associated with lower blood pressure. Since ischaemic stroke constitutes the majority of total stroke cases, the findings on intracerebral haemorrhage should not diminish the fundamental importance of high adiposity as a major modifiable determinant of overall stroke. In view of the substantial risks associated with high blood pressure, this study highlights the importance of controlling blood pressure (and other intermediate risk factors) for prevention of both ischaemic stroke and intracerebral haemorrhage, irrespective of levels of adiposity.
