# Supplementary material for: Adiposity and risk of ischaemic and haemorrhagic stroke in 0·5 million Chinese men and women: a prospective cohort study
Source: Lancet Glob Health. 2018 May 14;6(6):e630–40. doi: 10.1016/S2214-109X(18)30216-X (PMC5960068; doi:10.1016/S2214-109X(18)30216-X)

THE LANCET
Global Health

Supplementary appendix

This appendix formed part of the original submission and has been peer reviewed. We post it as supplied by the authors.

Supplement to: Chen Z, Iona A, Parish S, et al. Adiposity and risk of ischaemic and haemorrhagic stroke in 0·5 million Chinese men and women: a prospective cohort study. *Lancet Glob Health* 2018; **6:** e630–40.

Table of Supplemental Contents

Table of Supplemental Contents

Page 2. China Kadoorie Biobank collaborative group and members

Page 3. Supplemental Table S1. Correlations between different measures of adiposity at baseline among 175,894 men and 234,735 women.

Page 4. Supplemental Figure S1. Adjusted HRs for a)ischaemic stroke and b) intracerebral haemorrhage by usual SBP, stratified by age (left panel) and age and sex (right panel).

Page 5. Supplemental Table S2. Proportion of stroke attributable to high adiposity, using different BMI cut-points.

Page 6. Supplemental Figure S2. Adjusted HRs for a)ischaemic stroke and b) intracerebral haemorrhage by baseline BMI, in urban and rural areas.

Page 7. Supplemental Figure S3. Adjusted HRs for a)ischaemic stroke and b) intracerebral haemorrhage per 5 kg/m^2 higher baseline BMI by study areas.

Page 8. Supplemental Figure S4. Adjusted HRs for a)ischaemic stroke and b) intracerebral haemorrhage by baseline BMI, in never and ever regular smokers.

Page 9. Supplemental Figure S5. Adjusted HRs for a)ischaemic stroke and b) intracerebral haemorrhage per 5 kg/m^2 higher baseline BMI by baseline characteristics.

Page 10. Supplemental Table S3. Adjusted HRs for ischaemic stroke and intracerebral haemorrhage by baseline BMI, with stepwise exclusions.

Page 11. Supplemental Figure S6. Adjusted HRs for a) ischaemic stroke and b) intracerebral haemorrhage by baseline BMI, %BF and WC, with and without adjustment for mediators.

Page 12. Supplemental Table S4. Adjusted HRs for ischaemic stroke, intracerebral haemorrhage and total stroke associated with 1 SD difference in baseline adiposity measures, with and without adjustments for main mediators.

Page 13. Supplemental Table S5. Adjusted HRs for ischaemic stroke, intracerebral haemorrhage and total stroke associated with 1 SD difference in adiposity measures, with and without correction for regression dilution bias.

Page 14. Supplemental Figure S7. Adjusted HRs for a)ischaemic stroke and b) intracerebral haemorrhage by BMI at age 25 and BMI change, with and without adjustment for mediators

China Kadoorie Biobank collaborative group and members

International Steering Committee: Junshi Chen, Zhengming Chen (PI), Robert Clarke, Rory Collins, Yu Guo, Liming Li (PI), Jun Lv, Richard Peto, Robin Walters.

International Co-ordinating Centre, Oxford: Daniel Avery, Derrick Bennett, Ruth Boxall, Fiona Bragg, Yumei Chang, Yiping Chen, Zhengming Chen, Robert Clarke, Huaidong Du, Simon Gilbert, Alex Hacker, Michael Holmes, Andri Iona, Christiana Kartsonaki, Rene Kerosi, Om Kurmi, Sarah Lewington, Garry Lancaster, Kuang Lin, John McDonnell, Iona Millwood, Qunhua Nie, Jayakrishnan Radhakrishnan, Paul Ryder, Sam Sansome, Dan Schmidt, Paul Sherliker, Rajani Sohoni, Becky Stevens, Iain Turnbull, Robin Walters, Jenny Wang, Lin Wang, Neil Wright, Ling Yang, Xiaoming Yang.

National Co-ordinating Centre, Beijing: Zheng Bian, Ge Chen, Yu Guo, Xiao Han, Can Hou, Jun Lv, Pei Pei, Shuzhen Qu, Yunlong Tan, Canqing Yu.

10 Regional Co-ordinating Centres: Qingdao Qingdao CDC: Zengchang Pang, Ruqin Gao, Shaojie Wang, Yongmei Liu, Ranran Du, Yajing Zang, Liang Cheng, Xiaocao Tian, Hua Zhang. Licang CDC: Silu Lv, Junzheng Wang, Wei Hou. **Heilongjiang** Provincial CDC: Jiyuan Yin, Ge Jiang, Xue Zhou. Nangang CDC: Liqiu Yang, Hui He, Bo Yu, Yanjie Li, Huaiyi Mu, Qinai Xu, Meiling Dou, Jiaojiao Ren. **Hainan** Provincial CDC: Shanqing Wang, Ximin Hu, Hongmei Wang, Jinyan Chen, Yan Fu, Zhenwang Fu, Xiaohuan Wang. Meilan CDC: Min Weng, Xiangyang Zheng, Yilei Li, Huimei Li,Yanjun Wang. **Jiangsu** Provincial CDC: Ming Wu, Jinyi Zhou, Ran Tao, Jie Yang. Suzhou CDC: Chuanming Ni, Jun Zhang, Yihe Hu, Yan Lu, , Liangcai Ma, Aiyu Tang, Shuo Zhang, Jianrong Jin, Jingchao Liu. **Guangxi** Provincial CDC: Zhenzhu Tang, Naying Chen, Ying Huang. Liuzhou CDC: Mingqiang Li, Jinhuai Meng, Rong Pan, Qilian Jiang, Weiyuan Zhang, Yun Liu, Liuping Wei, Liyuan Zhou, Ningyu Chen, Hairong Guan. **Sichuan** Provincial CDC: Xianping Wu, Ningmei Zhang, Xiaofang Chen, Xuefeng Tang. Pengzhou CDC: Guojin Luo, Jianguo Li, Xiaofang Chen, Xunfu Zhong, Jiaqiu Liu, Qiang Sun. **Gansu** Provincial CDC: Pengfei Ge, Xiaolan Ren, Caixia Dong. Maiji CDC: Hui Zhang, Enke Mao, Xiaoping Wang, Tao Wang, Xi Zhang. **Henan** Provincial CDC: Ding Zhang, Gang Zhou, Shixian Feng, Liang Chang, Lei Fan. Huixian CDC: Yulian Gao, Tianyou He, Huarong Sun, Pan He, Chen Hu, Qiannan Lv, Xukui Zhang. **Zhejiang** Provincial CDC: Min Yu, Ruying Hu, Hao Wang. Tongxiang CDC: Yijian Qian, Chunmei Wang, Kaixue Xie, Lingli Chen, Yidan Zhang, Dongxia Pan. **Hunan** Provincial CDC: Yuelong Huang, Biyun Chen, Li Yin, Donghui Jin, Huilin Liu, Zhongxi Fu, Qiaohua Xu. Liuyang CDC: Xin Xu, Hao Zhang, Youping Xiong, Huajun Long, Xianzhi Li, Libo Zhang, Zhe Qiu.

Supplemental Table S1: Correlations* between different measures of adiposity at baseline among 175,894 men and 234,735 women

		WC	WHR	%BF	BMI25	Standing height	Sitting height
**BMI**	Male:	0.86	0.63	0.80	0.30	0.06	0.18
	Female:	0.84	0.54	0.89	0.34	0.03	0.13
**WC**	Male:		0.81	0.74	0.15	0.27	0.31
	Female:		0.79	0.79	0.23	0.19	0.20
**WHR**	Male:			0.59	0.13	0.02	0.04
	Female:			0.53	0.17	-0.04	-0.07
**%BF**	Male:				0.14	0.04	0.10
	Female:				0.25	-0.01	0.06
**BMI25**	Male:					-0.18	-0.11
	Female:					-0.14	-0.09
**Standing Height**	Male:						0.78
	Female:						0.77

BMI: body mass index; WC: waist-circumference; WHR: waist-hip ratio; %BF: Percentage body fat; BMI25: BMI at 25.

* Pearson partial correlation coefficients, adjusted for area and 5-year age group.

Supplemental Figure S1. Adjusted HRs for a) ischaemic stroke and b) intracerebral haemorrhage by usual SBP, stratified by age (left panel) and age and sex (right panel)

a) Ischaemic stroke
(25,210 events)

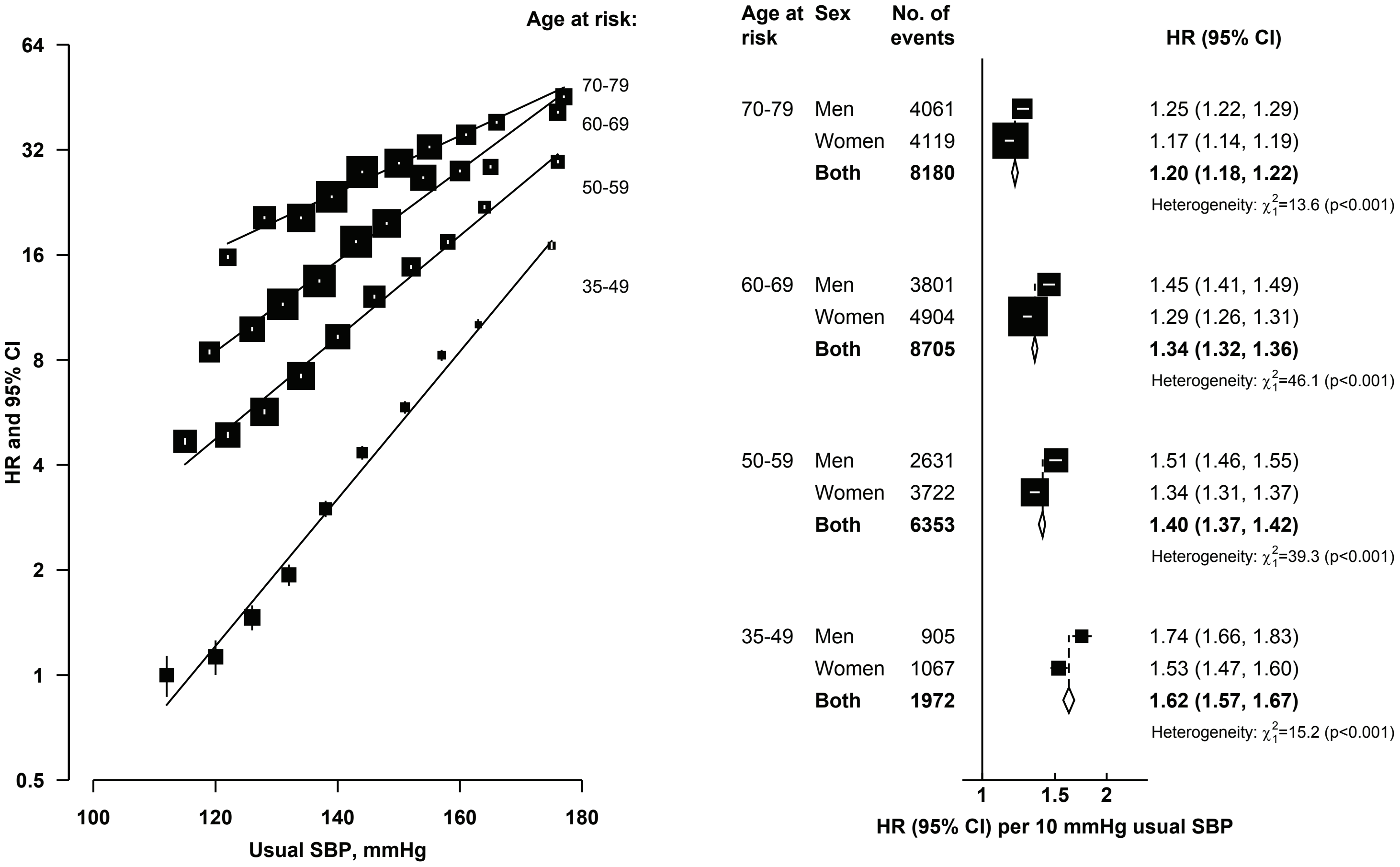

b) Intracerebral haemorrhage
(5,380 events)

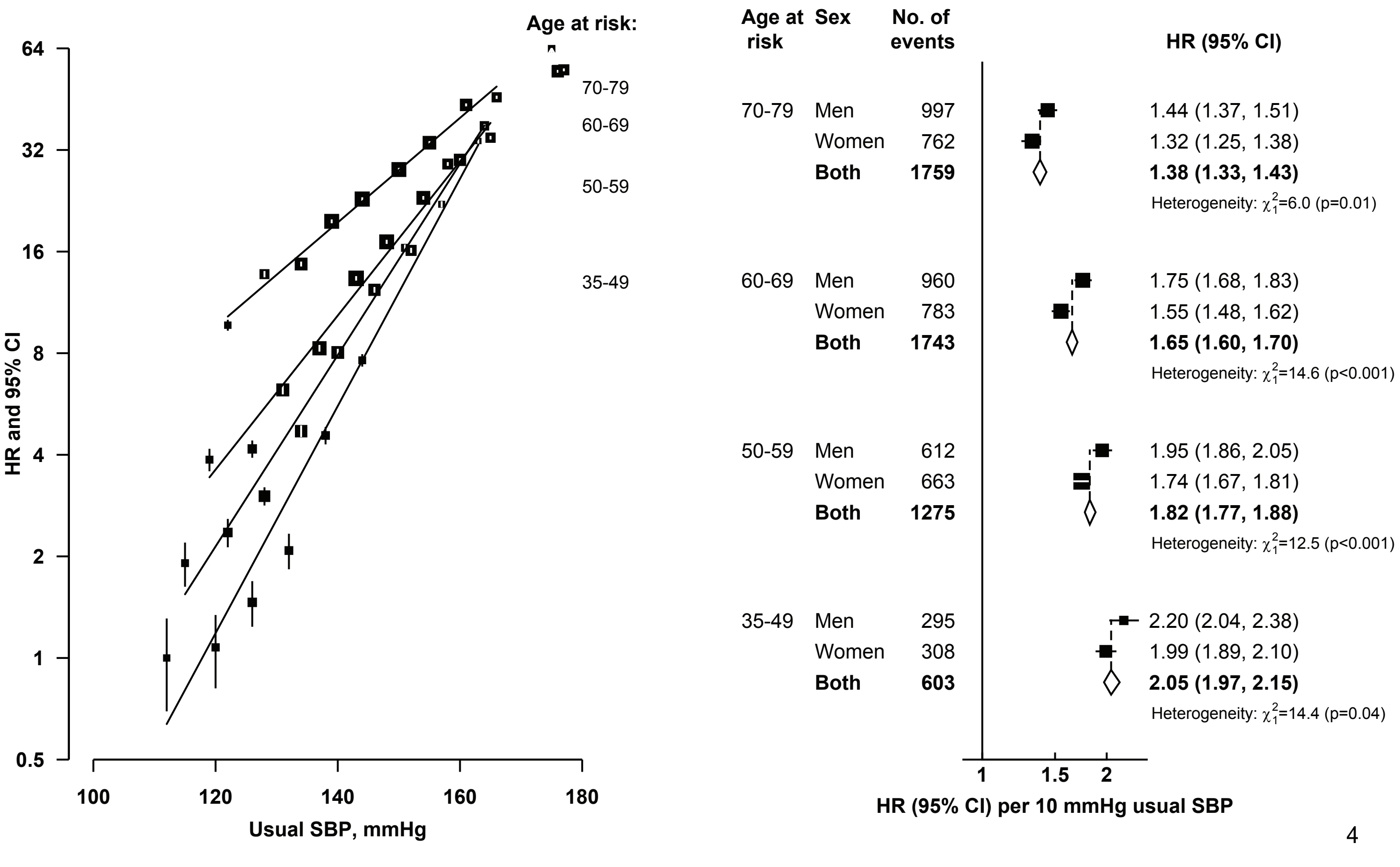

Supplemental Table S2: Proportion of stroke attributable to high adiposity, using different BMI cut-points

	No. of events	% of participants in each BMI category	HR*	PAF† (%)
**Ischaemic stroke**				
BMI >20 kg/m^2	18955	84.9	1.51	28.8
BMI >23 kg/m^2	13556	54.7	1.43	16.5
BMI ≥25 kg/m^2	9070	33.4	1.42	9.9
**Intracerebral haemorrhage**				
BMI >20 kg/m^2	3189	84.9	1.14	10.6
BMI >23 kg/m^2	1984	54.7	1.14	6.7
BMI ≥25 kg/m^2	1273	33.4	1.25	6.7
**All stroke**				
BMI >20 kg/m^2	23427	84.9	1.42	24.9
BMI >23 kg/m^2	16390	54.7	1.37	14.7
BMI ≥25 kg/m^2	10901	33.4	1.38	9.2

* Stratified by age, sex and study area, and adjusted for education, smoking, alcohol consumption, physical activity and self-rated health. The HRs were corrected for regression dilution bias using self-correlation coefficient of BMI between baseline and 1st resurvey (r_{BMI}=0.93).

† The fraction of events in the population that is attributable to adiposity (i.e., the population-attributable fraction; PAF) is P(HR-1) divided by RR, with P being the prevalence of adiposity, using Miettinen formula. (Reference: Rockhill B, Newman B, Weinberg C. Use and misuse of population attributable fractions. *Am J Public Health* 1998; 88: 15-19)

Supplemental Figure S2. Adjusted HRs for a) ischaemic stroke and b) intracerebral haemorrhage by baseline BMI, in urban and rural areas

i) Urban

a) Ischaemic stroke

(13,395 events)

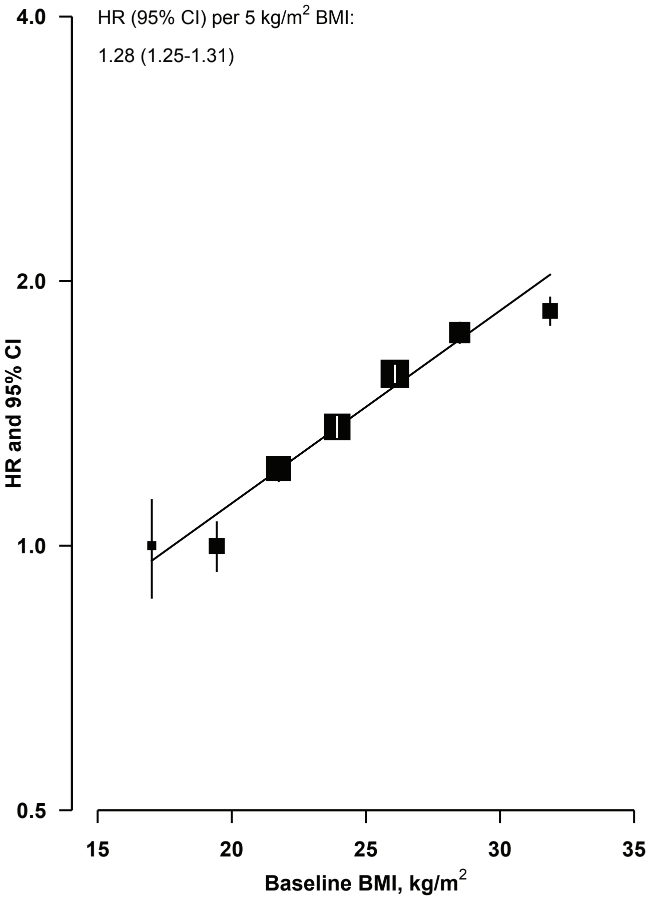

b) Intracerebral haemorrhage

(1,226 events)

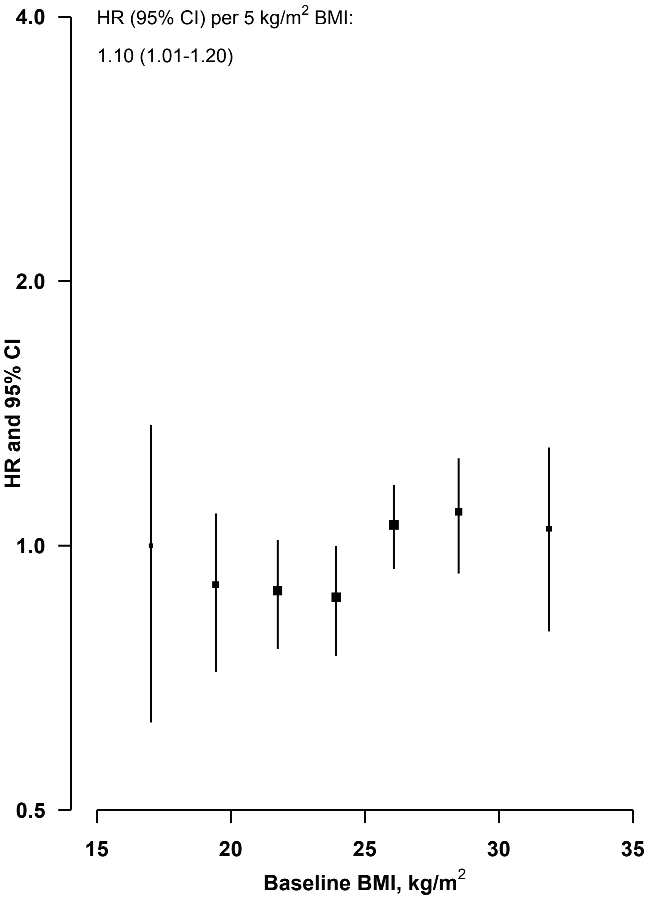

ii) Rural

a) Ischaemic stroke

(11,815 events)

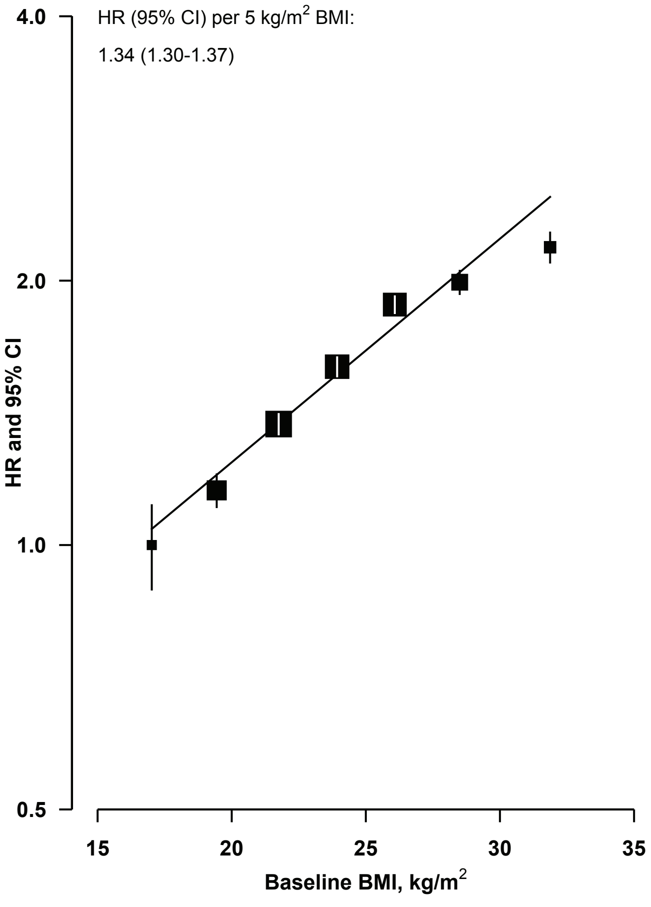

b) Intracerebral haemorrhage

(4,154 events)

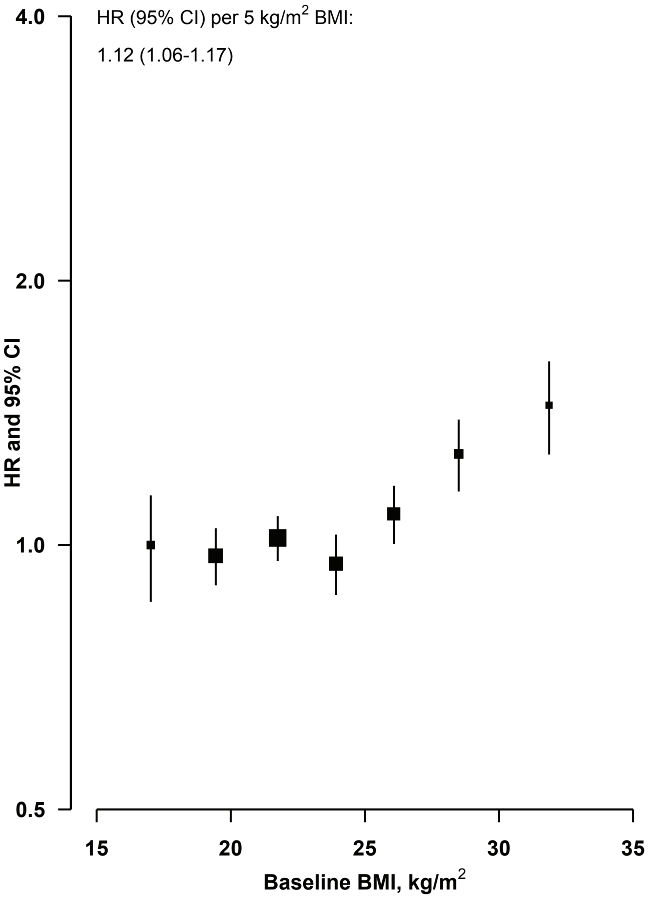

Supplemental Figure S3. Adjusted HRs for a) ischaemic stroke and b) intracerebral haemorrhage per 5 kg/m^2 higher baseline BMI by study areas

a) Ischaemic stroke

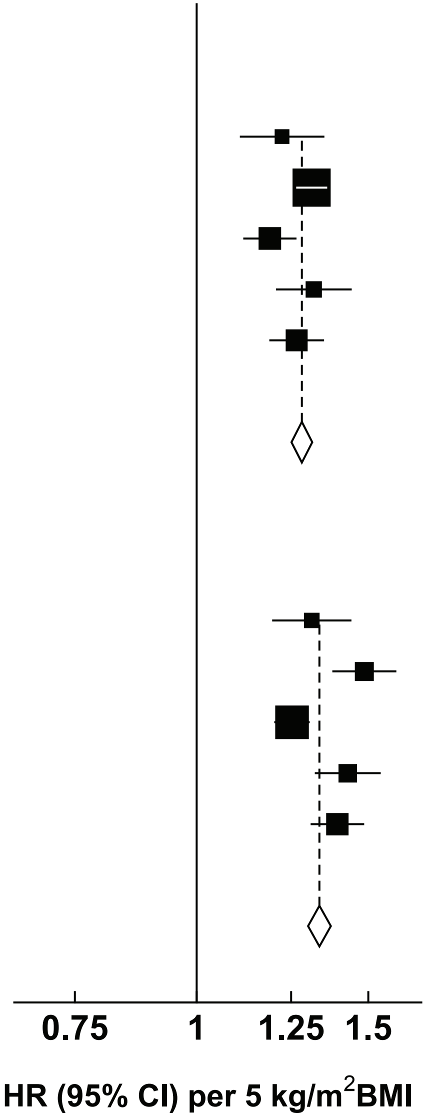

	No. of events	HR (95% CI)
**Urban**		
Qingdao	863	1.22 (1.11, 1.35)
Harbin	6713	1.31 (1.27, 1.36)
Haikou	2327	1.19 (1.12, 1.26)
Suzhou	1143	1.32 (1.21, 1.44)
Lizhou	2349	1.27 (1.19, 1.35)
		Heterogeneity: χ^2_4=9.1 (p=0.06)
**All**	**13395**	**1.28 (1.25, 1.31)**
**Rural**		
Sichuan	1059	1.31 (1.20, 1.44)
Gansu	1641	1.49 (1.38, 1.60)
Henan	4976	1.25 (1.20, 1.30)
Zhejiang	1555	1.43 (1.32, 1.54)
Hunan	2584	1.39 (1.31, 1.48)
		Heterogeneity: χ^2_4=23.4 (p<0.001)
**All**	**11815**	**1.34 (1.30, 1.37)**

b) Intracerebral haemorrhage

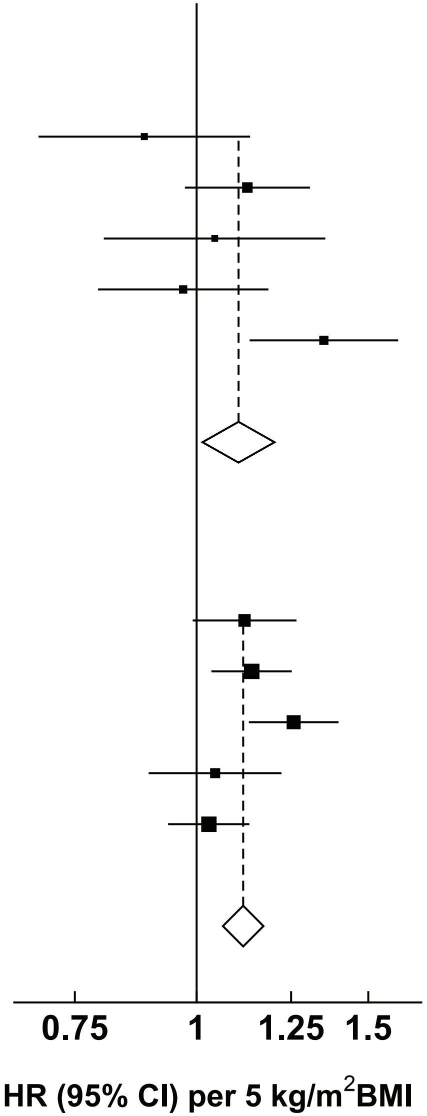

	No. of events	HR (95% CI)
**Urban**		
Qingdao	148	0.88 (0.69, 1.13)
Harbin	388	1.13 (0.97, 1.30)
Haikou	132	1.04 (0.80, 1.35)
Suzhou	246	0.97 (0.79, 1.18)
Lizhou	312	1.35 (1.14, 1.61)
		Heterogeneity: χ^2_4=10.2 (p=0.04)
**All**	**1226**	**1.10 (1.01, 1.20)**
**Rural**		
Sichuan	654	1.12 (0.99, 1.26)
Gansu	1103	1.14 (1.04, 1.25)
Henan	752	1.26 (1.13, 1.40)
Zhejiang	419	1.04 (0.90, 1.22)
Hunan	1226	1.03 (0.94, 1.13)
		Heterogeneity: χ^2_4=8.8 (p=0.07)
**All**	**4154**	**1.12 (1.06, 1.17)**

Supplemental Figure S4. Adjusted HRs for a) ischaemic stroke and b) intracerebral haemorrhage by baseline BMI, in never and ever regular smokers

i) Never smokers

a) Ischaemic stroke

(16,086 events)

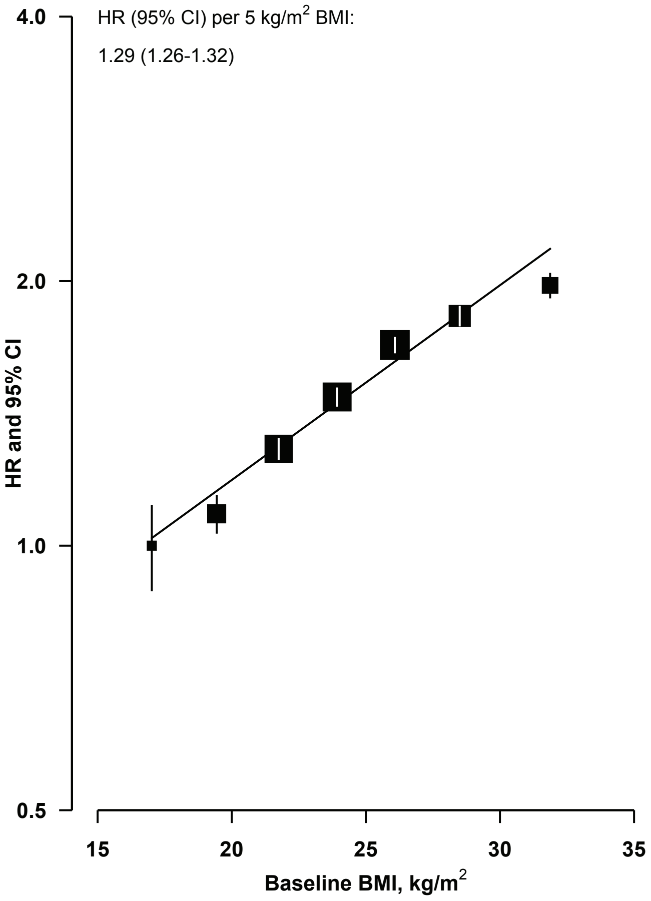

b) Intracerebral haemorrhage

(3,053 events)

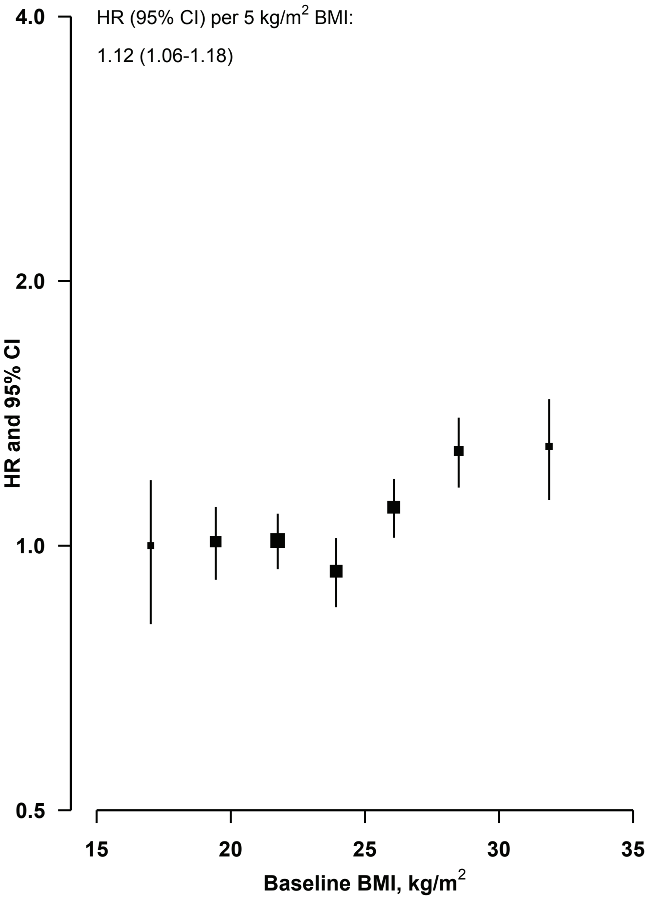

ii) Ever regular smokers

a) Ischaemic stroke

(9,124 events)

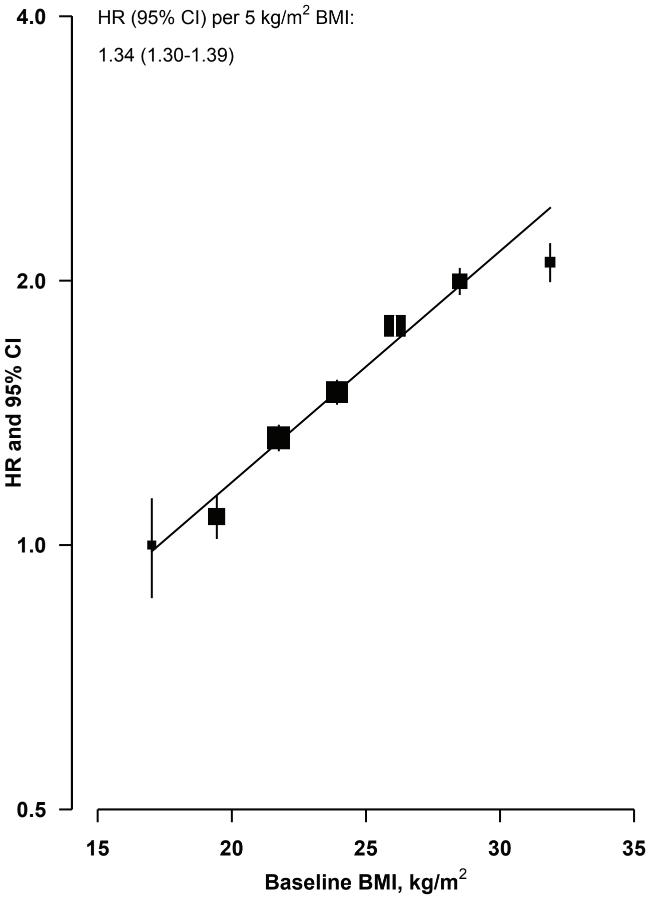

b) Intracerebral haemorrhage

(2,327 events)

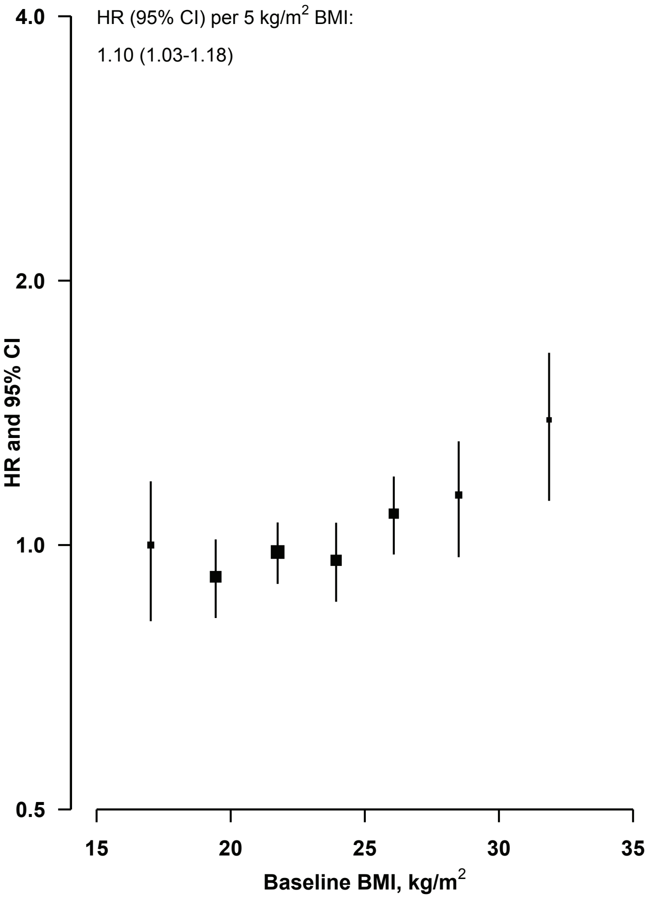

Supplemental Figure S5. Adjusted HRs for a) ischaemic stroke and b) intracerebral haemorrhage per 5 kg/m^2 higher baseline BMI by baseline characteristics

a) Ischaemic stroke

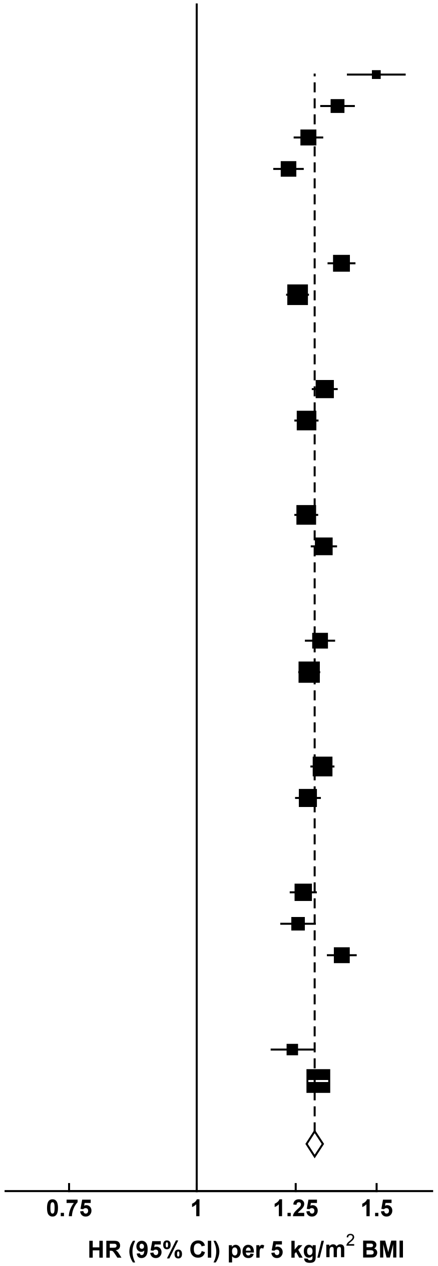

b) Intracerebral haemorrhage

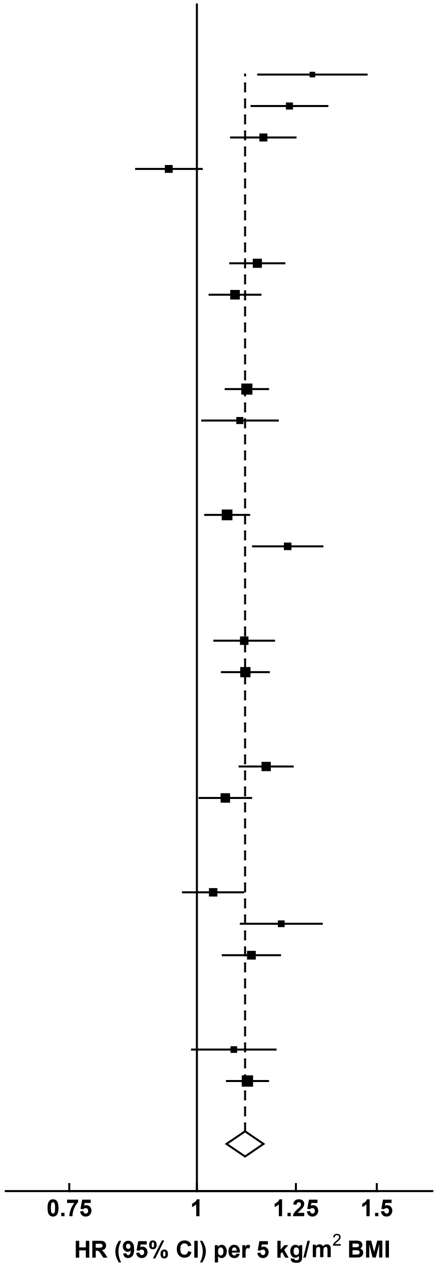

Characteristics	a) No. of events	a) HR (95% CI)	b) No. of events	b) HR (95% CI)
**Age at risk**				
35-49	1972	1.50 (1.41, 1.60)	603	1.30 (1.15, 1.47)
50-59	6353	1.37 (1.32, 1.42)	1275	1.23 (1.13, 1.34)
60-69	8705	1.29 (1.25, 1.33)	1743	1.16 (1.08, 1.25)
70-79	8180	1.23 (1.19, 1.27)	1759	0.94 (0.87, 1.01)
		Trend: χ^2_1=41.2 (p<0.001)		Trend: χ^2_1=30.3 (p<0.001)
**Gender**				
Men	11398	1.39 (1.35, 1.43)	2864	1.15 (1.08, 1.22)
Women	13812	1.26 (1.23, 1.28)	2516	1.09 (1.03, 1.15)
		Heterogeneity: χ^2_1=28.2 (p<0.001)		Heterogeneity: χ^2_1=1.3 (p=0.2)
**Region**				
Rural	11815	1.33 (1.30, 1.37)	4154	1.12 (1.07, 1.17)
Urban	13395	1.28 (1.25, 1.31)	1226	1.10 (1.01, 1.20)
		Heterogeneity: χ^2_1=5.0 (p=0.03)		Heterogeneity: χ^2_1=0.0 (p=0.8)
**Education, years**				
< 6	13548	1.28 (1.25, 1.31)	3839	1.07 (1.02, 1.12)
6+	11662	1.33 (1.30, 1.37)	1541	1.23 (1.14, 1.33)
		Heterogeneity: χ^2_1=4.8 (p=0.04)		Heterogeneity: χ^2_1=8.7 (p=0.004)
**Regular smoking**				
Ever	9124	1.32 (1.28, 1.36)	2327	1.11 (1.04, 1.19)
Never	16086	1.29 (1.26, 1.32)	3053	1.12 (1.06, 1.18)
		Heterogeneity: χ^2_1=1.5 (p=0.2)		Heterogeneity: χ^2_1=0.0 (p=0.6)
**Alcohol consumption**				
Ever	14151	1.33 (1.29, 1.36)	2765	1.17 (1.10, 1.24)
Never	11059	1.28 (1.25, 1.32)	2615	1.07 (1.01, 1.13)
		Heterogeneity: χ^2_1=1.1 (p=0.3)		Heterogeneity: χ^2_1=0.1 (p=0.8)
**Physical activity, Met-h/day**				
<10	10107	1.27 (1.24, 1.31)	1883	1.04 (0.97, 1.11)
10-14.9	5823	1.26 (1.21, 1.30)	1070	1.21 (1.10, 1.32)
15+	9280	1.39 (1.34, 1.43)	2427	1.13 (1.06, 1.21)
		Trend: χ^2_1=17.0 (p<0.001)		Trend: χ^2_1=3.6 (p=0.09)
**Self rated health**				
Poor	3187	1.24 (1.18, 1.30)	868	1.09 (0.99, 1.19)
Good/Excellent	22023	1.32 (1.29, 1.34)	4512	1.12 (1.07, 1.17)
		Heterogeneity: χ^2_1=5.1 (p=0.02)		Heterogeneity: χ^2_1=0.3 (p=0.6)
**All**	**25210**	**1.30 (1.28, 1.33)**	**5380**	**1.11 (1.07, 1.16)**

Supplemental Table S3: Adjusted HRs of ischaemic stroke and intracerebral haemorrhage by baseline BMI, with stepwise exclusions

Exclusions	No. of events	Categories of baseline BMI (kg/m^2)						
		<18.0	18.0 to <20.5	20.5 to <23.0	23.0 to <25.0	25.0 to <27.5	27.5 to <30.0	≥30.0
**Ischaemic stroke**								
any prior diseases*	13954	1.00 (0.90-1.11)	1.05 (1.00-1.10)	1.21 (1.17-1.26)	1.39 (1.34-1.44)	1.58 (1.52-1.64)	1.72 (1.64-1.81)	1.69 (1.56-1.83)
+ self-reported poor health status	12725	1.00 (0.89-1.12)	1.04 (0.99-1.09)	1.19 (1.15-1.23)	1.37 (1.32-1.42)	1.56 (1.51-1.62)	1.72 (1.63-1.82)	1.71 (1.57-1.86)
+ first three-years of follow-up	10061	1.00 (0.87-1.14)	1.08 (1.01-1.14)	1.21 (1.17-1.26)	1.40 (1.34-1.46)	1.60 (1.54-1.67)	1.80 (1.69-1.91)	1.80 (1.64-1.97)
+ ever smokers	6380	1.00 (0.84-1.19)	1.15 (1.07-1.24)	1.23 (1.17-1.30)	1.41 (1.34-1.49)	1.61 (1.53-1.70)	1.87 (1.75-2.01)	1.86 (1.67-2.07)
**Intracerebral haemorrhage**								
any prior diseases*	2908	1.00 (0.84-1.20)	1.01 (0.93-1.10)	1.02 (0.95-1.09)	0.97 (0.90-1.06)	1.06 (0.97-1.16)	1.15 (1.00-1.32)	1.24 (1.01-1.54)
+ self-reported poor health status	2587	1.00 (0.82-1.22)	1.07 (0.98-1.18)	1.03 (0.96-1.11)	1.00 (0.92-1.09)	1.09 (0.99-1.20)	1.17 (1.02-1.36)	1.27 (1.01-1.60)
+ first three-years of follow-up	1778	1.00 (0.78-1.28)	1.04 (0.93-1.16)	1.04 (0.95-1.13)	1.01 (0.92-1.12)	1.04 (0.93-1.17)	1.18 (1.00-1.41)	1.20 (0.90-1.59)
+ ever smokers	1013	1.00 (0.70-1.42)	1.21 (1.04-1.40)	1.17 (1.04-1.31)	1.04 (0.91-1.19)	1.10 (0.95-1.28)	1.27 (1.02-1.57)	1.21 (0.86-1.70)

* As well as CVD, the analyses further excluded those with prior history of self-reported physician-diagnosed cancer, chronic respiratory diseases, chronic renal diseases, peptic ulcer, chronic hepatitis and cirrhosis and gallbladder disease.

Supplemental Figure S6. Adjusted HRs for a) ischaemic stroke and b) intracerebral haemorrhage by baseline BMI, %BF and WC, with and without adjustment for mediators

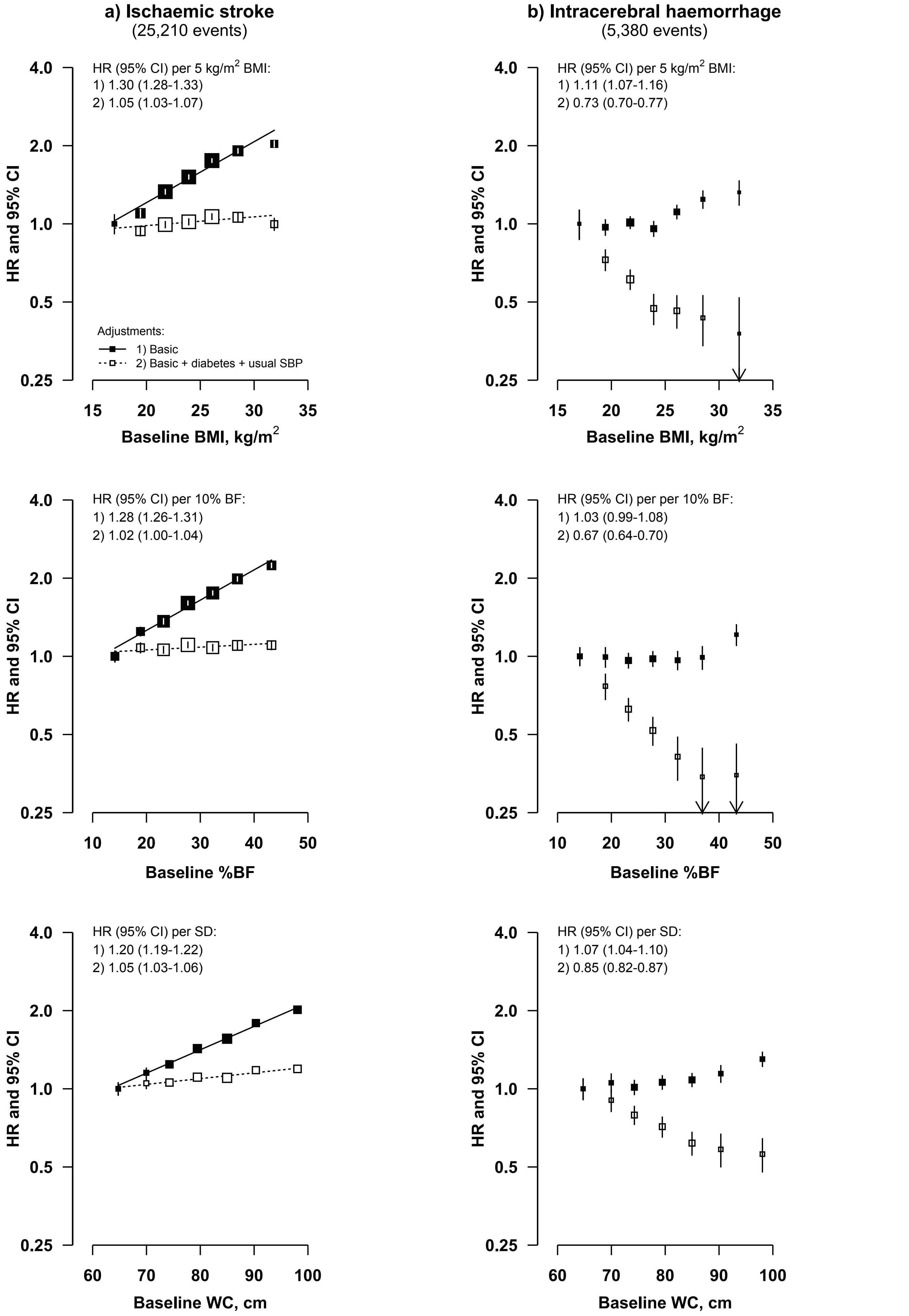

Supplemental Table S4: Adjusted HRs for ischaemic stroke, intracerebral haemorrhage and total stroke associated with 1 SD difference in baseline adiposity measures, with and without adjustments for main mediators

		Ischaemic stroke		Intracerebral haemorrhage		All stroke	
	**1 SD value**	**Model 1** *	**Model 2** †	**Model 1** *	**Model 2** †	**Model 1** *	**Model 2** †
		HR (95% CI)	HR (95% CI)	HR (95% CI)	HR (95% CI)	HR (95% CI)	HR (95% CI)
**BMI**	3.18	1.19 (1.18-1.21)	1.03 (1.01-1.04)	1.09 (1.06-1.12)	0.82 (0.79-0.85)	1.17 (1.16-1.19)	0.99 (0.98-1.00)
**WC**	8.97	1.21 (1.20-1.23)	1.05 (1.04-1.07)	1.09 (1.06-1.13)	0.86 (0.83-0.89)	1.19 (1.17-1.20)	1.01 (1.00-1.03)
**WHR**	0.06	1.19 (1.17-1.21)	1.06 (1.05-1.08)	1.13 (1.10-1.17)	0.95 (0.92-0.98)	1.18 (1.16-1.19)	1.04 (1.03-1.05)
**%BF**	6.42	1.20 (1.18-1.21)	1.02 (1.01-1.04)	1.04 (1.01-1.08)	0.77 (0.75-0.80)	1.16 (1.15-1.18)	0.97 (0.96-0.99)

* HRs were stratified by age, sex and area, and adjusted for education, smoking, alcohol drinking, physical activity and self-rated health status.
† HRs were additionally adjusted for usual SBP and diabetes status at baseline.

Supplemental Table S5: Adjusted HRs for ischaemic stroke, intracerebral haemorrhage and total stroke associated with 1 SD difference in adiposity measures, with and without correction for regression dilution bias

		Ischaemic stroke		Intracerebral haemorrhage		All stroke	
	**SD**	**Baseline** *	**Usual** †	**Baseline** *	**Usual** †	**Baseline** *	**Usual** †
		HR (95% CI)	HR (95% CI)	HR (95% CI)	HR (95% CI)	HR (95% CI)	HR (95% CI)
**BMI**	3.18	1.19 (1.18-1.21)	1.21 (1.19-1.23)	1.09 (1.06-1.12)	1.10 (1.06-1.13)	1.17 (1.16-1.19)	1.18 (1.17-1.21)
**WC**	8.97	1.21 (1.20-1.23)	1.26 (1.25-1.28)	1.09 (1.06-1.13)	1.11 (1.07-1.16)	1.19 (1.17-1.20)	1.23 (1.21-1.25)
**WHR**	0.06	1.19 (1.17-1.21)	1.31 (1.27-1.34)	1.13 (1.10-1.17)	1.21 (1.16-1.27)	1.18 (1.16-1.19)	1.29 (1.26-1.31)
**%BF**	6.42	1.20 (1.18-1.21)	1.25 (1.22-1.26)	1.04 (1.01-1.08)	1.05 (1.01-1.10)	1.16 (1.15-1.18)	1.20 (1.18-1.22)

* HRs were stratified by age, sex and area, and adjusted for education, smoking, alcohol drinking, physical activity and self-rated health status.

† HRs were additionally corrected for regression dilution bias using self-correlation coefficients between baseline and 1st resurvey (r_{BMI}=0.93, r_{WC}=0.83, r_{WHR}=0.65, $r_{\%BF}$=0.83).

Supplemental Figure S7. Adjusted HRs for a) ischaemic stroke and b) intracerebral haemorrhage by BMI at age 25 and BMI change, with and without adjustment for mediators

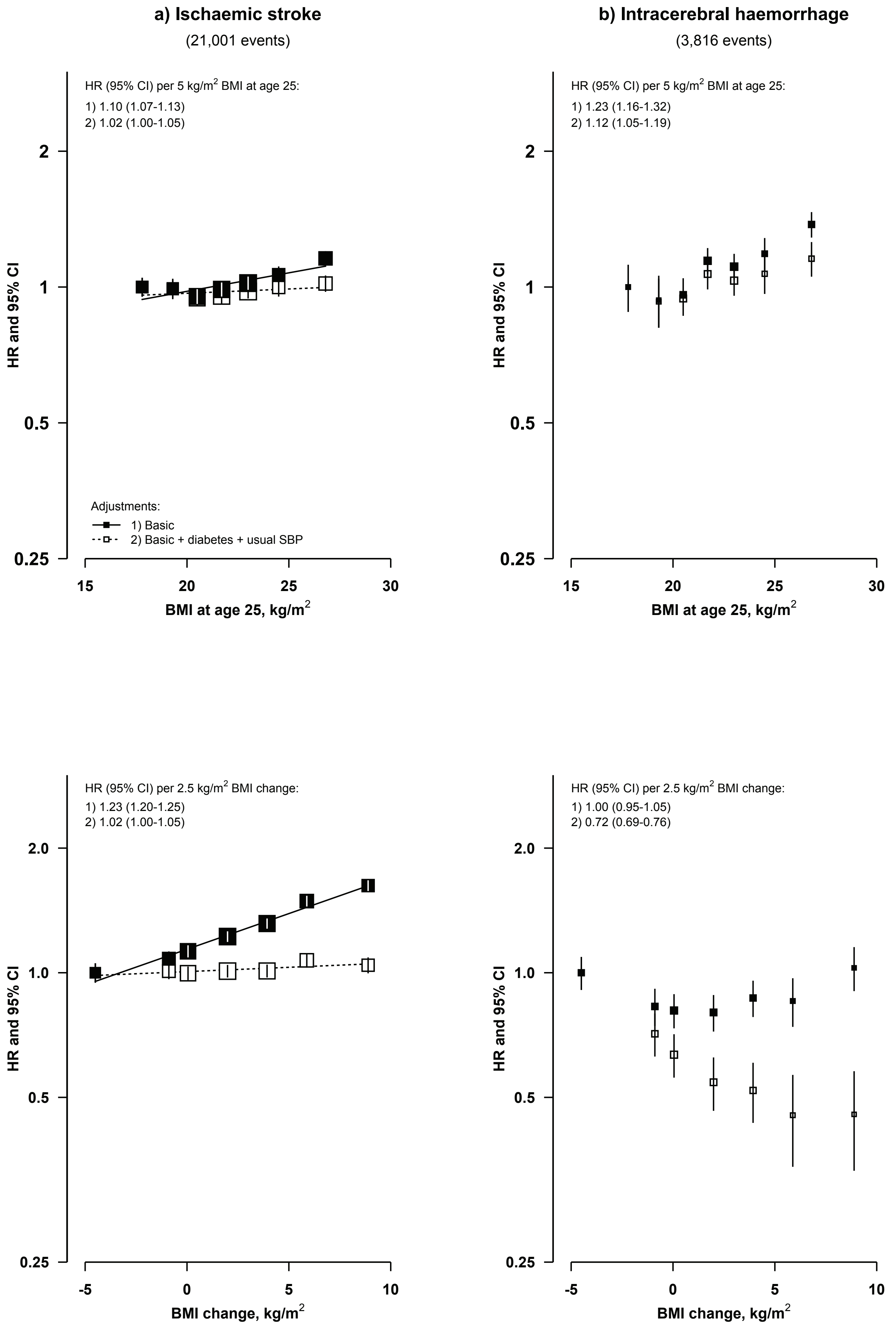

Supplement: Supplementary appendix [file mmc1.pdf]
